# SNP and indel frequencies at transcription start sites and at canonical and alternative translation initiation sites in the human genome

**DOI:** 10.1371/journal.pone.0214816

**Published:** 2019-04-12

**Authors:** Kerstin Neininger, Tobias Marschall, Volkhard Helms

**Affiliations:** 1 Center for Bioinformatics, Saarland University, 66123 Saarbrücken, Germany; 2 Graduate School of Computer Science, Saarland University, 66123 Saarbrücken, Germany; 3 Max Planck Institute for Informatics, 66123 Saarbrücken, Germany; University of Helsinki, FINLAND

## Abstract

Single-nucleotide polymorphisms (SNPs) are the most common form of genetic variation in humans and drive phenotypic variation. Due to evolutionary conservation, SNPs and indels (insertion and deletions) are depleted in functionally important sequence elements. Recently, population-scale sequencing efforts such as the 1000 Genomes Project and the Genome of the Netherlands Project have catalogued large numbers of sequence variants. Here, we present a systematic analysis of the polymorphisms reported by these two projects in different coding and non-coding genomic elements of the human genome (intergenic regions, CpG islands, promoters, 5’ UTRs, coding exons, 3’ UTRs, introns, and intragenic regions). Furthermore, we were especially interested in the distribution of SNPs and indels in direct vicinity to the transcription start site (TSS) and translation start site (CSS). Thereby, we discovered an enrichment of dinucleotides CpG and CpA and an accumulation of SNPs at base position −1 relative to the TSS that involved primarily CpG and CpA dinucleotides. Genes having a CpG dinucleotide at TSS position -1 were enriched in the functional GO terms “Phosphoprotein”, “Alternative splicing”, and “Protein binding”. Focusing on the CSS, we compared SNP patterns in the flanking regions of canonical and alternative AUG and near-cognate start sites where we considered alternative starts previously identified by experimental ribosome profiling. We observed similar conservation patterns of canonical and alternative translation start sites, which underlines the importance of alternative translation mechanisms for cellular function.

## Introduction

Polymorphic sites in the genome are a major source of phenotypic variation in human populations [1–3]. These polymorphisms are caused by random mutational processes such as nucleotide misincorporation during DNA replication, DNA damage, or erroneous activity of DNA-processing enzymes [4, 5]. These mutational forces are typically counteracted by DNA repair mechanisms [6] and by Darwinian selection [7]. In general, there exist two major types of sequence variations, transition and transversion SNPs, as well as insertions and deletions (indels). Thereby, transitions (mutation from C to T, or G to A on the second strand), are the most frequent substitution found [8]. Here we considered data from two large-scale sequencing projects that characterized the variability of human genome sequences: the 1000 Genomes Project (1000G) [9] and the Genome of the Netherlands Project (GoNL) [10, 11]. While the 1000G reconstructed genomes from 2,504 individuals from 26 populations, mostly from low-coverage data, the GoNL focused on 250 Dutch parent-offspring families sequenced at higher coverage, which additionally allowed to identify *de novo* mutations [11, 12]. Venter and colleagues recently presented an analysis of variations in 10,000 human genomes [13]. They investigated and reported different SNP densities for protein coding, RNA coding and gene regulatory elements. Castle analyzed SNPs in different genomic mRNA regions of human and mouse such as the start site, splice sites and miRNA binding sites and reported an anti–correlation between the number of SNPs and sequence conservation [14]. Tatarinova *et al*. used variants provided by the 3,000 Rice Genomes Project and investigated their genome–wide distribution [15].

The start site flanking region of translated sequences was shown to play a crucial role in translation initiation and is important for the recognition of the start sites by the ribosome scanning complex [16–19]. Marilyn Kozak found that a purine at position −3 and guanine at position +4 are crucial for efficient translation initiation [16, 17]. Noderer *et al*. analyzed the influence of all possible translation initiation starts between positions −6 and +5 using FACS-seq. They confirmed the high influence of the sequence context in direct vicinity of the start site on translational efficiency [18]. It was shown that SNPs in and around translation start sites can have a strong influence on the efficiency of the start site recognition and thus the translation machinery. For example, mutations in the translational start site of protein KLHL24 resulted in a shortened polypeptide due to ribosomal read-through of the mutated start site and initiation at an in-frame downstream AUG-Methionine [19].

Beside the canonical AUG, other codons that differ from AUG by one nucleotide were shown to also function as translation start sites [20, 21]. These so-called alternative start sites mainly occur in the 5’ UTR, but also in the coding DNA sequence (CDS), or the 3’ UTR [22]. Dependent on the location of a 5’ UTR alternative start site relative to the annotated start site, translation can be in-frame or out-of-frame and therefore result in, for instance, small upstream ORFs, elongated proteins, or alternative proteins [23]. Alternative translation initiation, and thus the resulting alternative proteins, are involved in regulatory processes and can be targeted to different cell compartments [24–26]. Ribosome profiling is an experimental technique to determine (alternative) translation start sites [21, 27–29]. A prediction model called *PreTIS* was developed by us to assist in the analysis of 5’ UTR sequences and reveal alternative non-AUG reading-frame-independent translation start sites in human and mouse [30].

**Fig 1:**
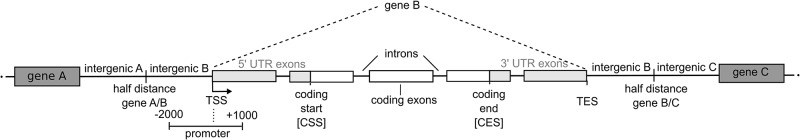
Definition of human genomic regions. Definition of the basic genomic regions: intergenic region, promoter region, 5’ UTR, coding exon(s), 3’ UTR, all exons, intron(s), intragenic region and CpG islands (not shown here). Shown is the + strand, − strand is analogous.

**Fig 2:**
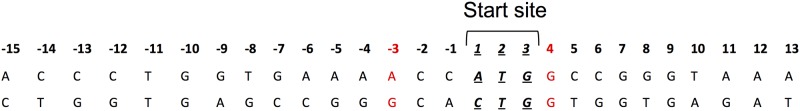
Sequence context around translation start sites. Two example sequence contexts are shown to visualize the definition of the flanking sequence around translation start sites. Per definition, positions around the translation start site are given relative to the start codon, which is denoted as 1,2 and 3. Position zero is left out. Positions -3R (R = purine) and +4G were shown to be crucial for translation initiation [16, 17] and are therefore highlighted in red color.

In this study, we carried out a systematic analysis of transition and transversion SNPs as well as indels that occur in nine types of coding and non-coding genomic elements in the human genome. We distinguished intergenic regions, CpG islands, promoters, 5’ UTRs, coding exons, 3’ UTRs, all exons, introns and intragenic regions, see Fig 1. Thereby, CpG islands can overlap with other categories such as promoter regions. Moreover, all exons, coding exons or intragenic regions overlap as well. Thus, these categories are non-exclusive. As primary datasets, we used SNPs and indels reported in the European cohort of the 1000 Genomes Project [9] and the GoNL project [10, 11]. Both datasets were analyzed separately. To test for neutrality, we applied the widely-used Tajima’s D statistic to different genomic elements [31]. We mainly focussed on the SNP pattern around transcription and translation initiation start sites since these SNPs may have direct effects on gene transcription and protein translation. Special attention was given to the translational flanking region that was defined as a window ranging from −15 to +13 with respect to the start codon, see Fig 2. With the common assumption that conservation reflects functional relevance, we compared the SNP distribution of canonical start sites and experimentally detected [21] alternative start sites in the 5’ UTR.

## Results and discussion

The primary focus of this study was to investigate SNPs and indels frequencies at transcription and translation start sites. Furthermore, we compared canonical with alternative translation start sites, detected by ribosome profiling, to shed light onto translational regulatory complexity. Before addressing these specific points, we will compare the results of our analysis of data from the 1000G and GoNL sequencing projects with the current literature.

### Distribution of different SNP and indel types on autosomes

For this study, we selected SNPs and indels reported by (a) the European cohort of the 1000G project (23,938,159 variants remained after filtering), and (b) the GoNL project (20,706,633 variants remained after filtering). In total, 63% of the annotated 1000G variants on human autosomes are transition SNPs resulting from deamination (and tautomerization) whereas 30% are transversion SNPs. The remaining variants (7%) are indels. In GoNL, the distribution is similar with 65%, 29% and 6% for transition, transversion, and indels, respectively. This is consistent with the 2:1 transition/transversion ratio.

### Variant distribution in nine sequence elements

Since different DNA elements such as CpG islands, 5’ UTRs, protein-encoding exons or intergenic regions may exhibit different patterns of sequence conservation, we separately investigated these elements in the 16,604 RefSeq genes that remained after filtering. First, genes that are located within other genes (complete overlap) were removed prior to further analysis (about 3%). Subsequently, about 5% of all remaining genes were removed due to staggered overlaps.

**Fig 3:**
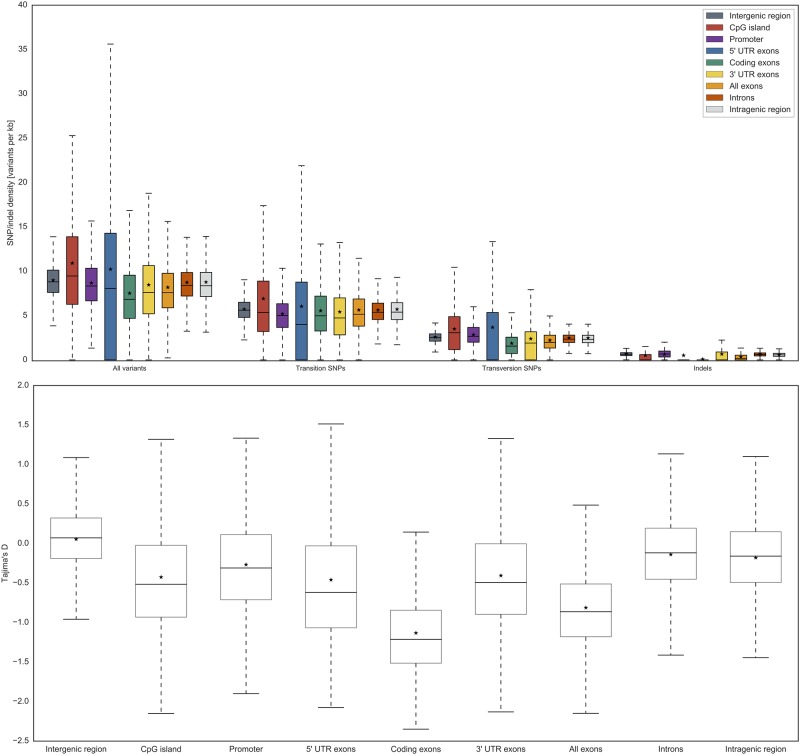
SNP and indel densities for all variant types and genomic elements considering 1000G data (European cohort). Upper panel: distribution of SNP and indel densities, the horizontal line (−) represents the median value, the asterisk (⋆) denotes the mean value. In total, the 1000G European super population comprises 503 individuals; Lower panel: Tajima’s D statistic was applied to evaluate the neutral evolution hypothesis.

We used SNP density (SNPs per kb) and Tajima’s D statistic [31] to compare the variant distribution of the different genomic elements with each other. Fig 3 illustrates the results for all SNP and indel types from 1000G data. The results for the GoNL data are shown in S1 Fig.

Considering 1000G data, median SNP densities were ∼ 8−9 SNPs per kb for each genomic element and all variant types, see Fig 3 (upper panel, leftmost group). Considering GoNL data, median SNP densities were on average slightly lower (∼ 6−7 SNPs per kb) compared to 1000G for each genomic element and all SNP types, see S1 Fig.

Fig 3 shows that protein-coding regions are conserved with a median SNP density of about 7 SNPs/kb for all SNP types. This is in accordance with earlier findings [13]. The boxplot for the 5’ UTR contains some outliers with a maximum SNP density of up to about 35 SNPs per kb for 1000G data, (see Fig 3, upper panel). This effect is due to the short 5’ UTR length of 230 bp on average (median 180 bp), see S2 Fig. In general, data from 1000G and GoNL provided similar results, see Fig 3 and S1 Fig.

Indels are especially rare in coding exons, with a mean SNP density of 0.09 (two-tailed Wilcoxon rank sum test, *p* < 1.4 × 10^−3^, see Fig 3 (upper panel, rightmost group), S1 and S4 Tables), since this type of mutation can cause frameshifts in the translated protein. Indel densities were significantly lower than SNP densities (two-tailed Wilcoxon rank sum test, *p* < 1.4 × 10^−3^, see S1 and S4 Tables). Especially CpG islands, 5’ UTRs, protein-encoding exons and 3’ UTRs showed a low amount (median: 0.0) of indels, see Fig 3 (upper panel, rightmost group), S1 and S4 Tables. An overview of all SNP and indel densities as well as the results of the statistical analyses can be found in S1 to S6 Tables. A decrease of deletions upstream of the transcription start site has been described in the literature [32]. To our knowledge, a similar effect has not been described yet for CpG islands. Indels might have more severe effects on transcription factor binding sites than base exchanges [33]. Hence, the low frequency of indels in CpG islands might be related to a strict conservation of functional sequences within this genomic (regulatory) element especially in CpG islands in the promoter regions of the mammalian genes [34].

Fig 3 (lower panel) shows Tajima’s D values for all genomic elements. Tajima’s D values < 0 point to a high number of rare alleles based on a growth in population size and/or purifying selection while values > 0 indicate a high number of alleles with average frequency. Intergenic regions (median: 0.07) were more or less neutral with values around 0. The smallest Tajima’s D values were found in coding exons (median: −1.21), followed by all exons (median: −0.86), 5’ UTR exons (median: −0.62), CpG islands (median: −0.52), and 3’ UTR exons (median: −0.49). Thus, our findings of smaller SNP densities (two-tailed Wilcoxon rank sum test, *p* < 1.4 × 10^−3^, see S1 and S4 Tables) in genetically important gene regions such as coding exons or 5’ UTRs are compatible with purifying selection to preserve their functionality. A high conservation of protein-coding regions and a lower conservation of intergenic regions and introns were reported before [13]. Especially, splice sites, i.e. the exon-intron boundaries, were shown to be highly conserved [13].

In summary, we obtained a similar picture on the conservation of genomic elements when either applying Tajima’s D statistic or calculating simple SNP densities (Fig 3) that is in good agreement with the findings reported by [13]–[15]. Slight differences were observed when comparing CpG islands with intergenic regions: the SNP density is on average higher in CpG islands (∼9 vs. ∼11 SNPs per kb, see Fig 3, upper panel) while the respective Tajima’s D index is smaller (median: −0.52 vs. 0.07, see Fig 3, lower panel). Moreover, considering 1000G and GoNL data provided similar SNP distributions.

The most polymorphic part of the human genome, the major histocompatibility complex (MHC) encodes over 160 proteins of diverse function including the HLA class I and II genes. These genes encode cell-surface proteins that bind peptide antigens, engage T cell receptors, and stimulate the T cell arm of adaptive immunity [35]. Hence, as expected, three such HLA genes also had the largest SNP density in the concatenated coding exon regions and two HLA genes in the promoter regions (-2000 bp to +1000 bp around the TSS) based on the 1000G data. S7 and S8 Tables list the Top100 genes with the largest SNP densities from the European cohort of the 1000G set. The advantage of a large whole-genome sequencing project is that sequence variations in all assembled genomic regions are covered to the same extent, whereas large SNP assemblies such as dbSNP reflect historic biases, the biomedical relevance of certain gene classes and other coincidences.

To illustrate such effects, we compared the genes encoding the two cytochrome p450 proteins CYP2A7 and CYP2D6 to the three mentioned HLA genes. The two CYP genes are 1.78 longer (1484, 1341 nt) than the HLA genes (810, 768, 801 nt). However, the 1000G data contains only 40 and 35 SNPs for the CYPs vs 71, 67 and 68 SNPs for the HLAs, respectively. This yields a fraction of 0.54. On the other hand, the GeneCards repository (https://www.genecards.org/) currently reports for HLA-DQB1 a total number of 6313 SNPs and 546 when filtered to “coding_sequence_variants”, 5509 and 316 for HLA-DQA1, and 6633 and 507 for HLAD-RB5. In contrast, for CYP2A7 GeneCard reports a total of 3872 SNPs out of which 692 SNPs are “coding_sequence_variants”, and 3074 and 909 for CYP2D6, respectively. These numbers are 1.75 times higher than for the HLA genes. By comparing the two ratios, it follows that GeneCards reports 3.2 times more exonic SNPs than 1000G for the two CPYs relative to HLAs. Although this is a very small-scale comparison, one can expect comparable effects between other gene classes. Whether this is after all a desirable or undesirable effect will depend on the particular research question.

### SNP and indel frequencies around the TSS and the CSS

SNPs and indels in the promoter regions and 5’ UTRs may have direct effects on gene transcription and protein translation. Thus, we investigated SNP densities and distributions around the transcription start site (TSS) and the translation/coding start site (CSS) separately and in more detail.

#### TSS—Transcription start site

**Fig 4:**
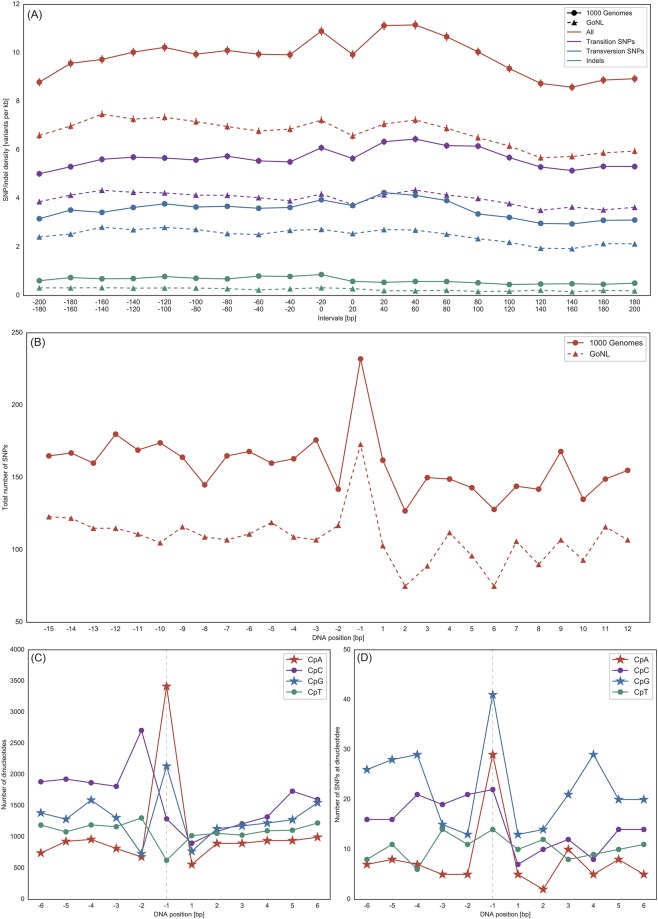
SNP and dinucleotide distribution around the TSS. A: Average SNP and indel density (1000G and GoNL data) around the TSS (±200 bp) of all RefSeq genes. The standard error of the mean is visualized for every datapoint. B: SNP pattern in direct vicinity (−15 to +12) of the TSS considering 1000G and GoNL data. Position 1 denotes the first intragenic nucleotide. C: Distribution of dinucleotides starting with cytosine in the flanking region of the TSS of all RefSeq genes. CpG and CpA dinucleotides are prevalent. D: Number of SNPs (1000G data) at individual dinucleotides. The majority of SNPs resides at CpG dinucleotides.

Fig 4A shows the local SNP density around the TSS in a range of ±200 bp around the TSS. Both, 1000G and GoNL data show a decrease in SNP density directly at the TSS. Moreover, there is a SNP density increase downstream of the TSS. This suggests that especially the region flanking the TSS (first few bases) on the downstream site, are especially important. This effect is also displayed in Fig 4B at base pair resolution. The same pattern was observed before [13, 36]. Also, the indel frequency decreased slightly at the TSS as indels might perturb protein-coding regions more strongly compared to base exchanges. Nevertheless, the number of indels is in general low such that we can only observe a slight indel depletion towards the TSS.

Next, we analyzed SNPs in direct vicinity to the TSS, see Fig 4B. The number of genes with at least one SNP in this sequence window is given in Table 1. In general, the number of SNPs directly downstream of the TSS is lower than directly upstream. Clearly noticeable is the high peak at position −1. To analyze statistical significance, we resampled the intervals and recorded the 1000 highest peaks within the shuffled intervals. Mean, median and standard deviation values amounted to 151.2, 151.0 and 5.8 (1000G) as well as 134.4, 134.0 and 5.6 (GoNL). As the reported peak at position −1 is higher (see Fig 4B), we define the increase at position −1 as significant.

**Table 1:** Number of start sites and SNPs in direct vicinity of TSS and CSS.

	TSS		CSS			
	RefSeq		RefSeq		HEK293	
	1000 Genomes	GoNL	1000 Genomes	GoNL	1000 Genomes	GoNL
Start sites harboring SNPs	3,777	2,681	3,319	2,277	1,276	838
SNPs in these start sites	4,472	3,043	3,819	2,519	1,487	942

We considered RefSeq and HEK293 genes as well as SNPs from the 1000G and GoNL project. Thereby, we investigated genes with at least one SNP in this sequence window. For the CSS, we considered AUG as start site for the RefSeq dataset and alternative AUG and all near-cognate codons for the HEK293 dataset.

The results of a recent study [13] also indicate an increase of mutations upstream of the TSS, although the window size utilized there does not allow further specification of the affected DNA positions. We observed that especially position −1 relative to the TSS is more frequently mutated than the surrounding positions. It was recently reported that the mutation rate in human genomes is elevated at protein-bound DNA sites such as active transcription factor binding sites or nucleosome positions [37]. This was shown to be due to the interference of the nucleotide excision repair (NER) machinery and DNA-binding proteins that results in a decreased NER activity [37]. Thus, one might speculate that DNA-bound transcription factors could block repair enzymes resulting in higher mutation frequencies at these positions. However, we observed that only the position −1 deviates from the general SNP pattern. Thus, we analyzed the underlying DNA sequence at and around position −1 in more detail, also keeping in mind that some polymorphisms can occur more frequently (e.g. in the context of methylated CpGs) compared to other dinucleotides.

Fig 4C displays the dinucleotide distribution around the TSS while Fig 4D denotes the respective SNP distribution for individual dinucleotides. Fig 4C shows that the frequency of CpG dinucleotides (and CpA dinucleotides, see below) is increased at position −1 compared to the surrounding positions. A CpG at position −1 means that the C is located at position −1 while the respective G resides at position 1. In general, CpG dinucleotides are enriched around the TSS and peak with the TSS, see S3 Fig. This symmetric CpG pattern was reported before [34]. 2,136 out of 16,603 considered RefSeq genes (one sequence context was removed due to the occurrence of “N”, i.e. an unknown base, in the hg19 reference genome) exhibited a CpG dinucleotide at position −1 corresponding to ∼13% of all genes. This number is twofold higher than expected from the dinucleotide frequencies observed in the human genome [38] and about 50% higher than at the neighboring positions (see Fig 4C). CpA dinucleotides were found at positions −1/+1 for 3,415 out of 16,603 genes amounting to 21% of all RefSeq genes (threefold higher than expected). The distribution of all 16 dinucleotides in the enlarged window (–15 to +12) is shown in S4 Fig.

Fig 4D displays the underlying SNP distribution (1000G data) for individual dinucleotides. At position −1, SNPs are most often found at CpG dinucleotides, followed by CpA dinucleotides. Thus, although there are more CpA than CpG dinucleotides at position −1 (see Fig 4C), SNPs are more frequently found in CpG context (ratios of $\frac{41}{2,136}=0.02$ and $\frac{29}{3,415}=0.008$, respectively). Rahbari et al. [39] found a higher proportion of point mutations at CpG sites in case of a C/T mutation. The frequencies for all 16 dinucleotides together with the underlying SNP pattern when considering 1000G and GoNL data are visualized in S5 and S6 Figs, respectively.

The emergence of CpG (and CpA) dinucleotides especially at TSS position −1 was reported before [40, 41] and this partially explains the higher mutation rate detected at this position. Fig 4B shows that the total number of SNPs in 1000G data increases from about 160 per position to 230 at position −1. According to Fig 4D about 20 SNPs of this increase are found at CpGs and 20–25 SNPs at CpAs. The manifestation of polymorphisms at CpG sites, where cytosine is often methylated, is influenced by epigenetic marks [42]. Since deamination of 5-methylcytosine results in thymine which is a regular base of DNA, the resulting G–T mismatches are less efficiently corrected than G–U mismatches [8, 43]. It has been proposed that the methylation of cytosines is one cause of the general CpG depletion of vertebrate genomes, where only unmethylated CpG islands tend to have a CpG content that reflects the frequency of cytosine and guanine in the genome [44]. The evolutionary stability of CpG islands has been related to a high selective pressure on these regions, especially on CpG islands in the promoter regions of the mammalian genes [34]. As shown in Fig 4C, CpA dinucleotides are also highly enriched at position −1. Interestingly, non-CpG methylation was reported for different mammalian cell types (e.g. embryonic stem cells but also differentiated cells) and it has been suggested that methylation in non-CpG context is involved in the regulation of gene expression [45–47]. Beside CpG sites, methylation is most often found at CpA sites compared to CpT and CpC sites [45].

Finally, we analyzed the 2,136 genes with a CpG dinucleotide at position −1 by a GO-term enrichment analysis using the DAVID resource (version 6.8) [48]. As background gene set, we used all RefSeq genes considered here. We applied DAVID default functional terms, Benjamini-Hochberg correction and an EASE score threshold (corresponding to a modified Fisher exact p-value) of 0.05. The results of the functional annotation are displayed in Table 2. It is noteworthy that more than half of the inspected 2,136 genes are associated with the terms “Phosphoprotein”, “Alternative splicing” and “Protein binding”, see Table 2. Another highly significant functional term was “Acetylation”. A significant enrichment of the GO term “(protein) phosphorylation” was also observed in rice for genes with decreased sequence conservation (variable genes) compared to other gene families such as transcription regulators (conserved genes) [15].

**Table 2:** Functional annotation of genes with a CpG at TSS position −1.

	Term	# Genes	% Genes	q–value
1.	Phosphoprotein	1110	52.0	3.5 ⋅ 10^−13^
2.	Acetylation	507	23.7	2.1 ⋅ 10^−9^
3.	Alternative splicing	1314	61.5	1.6 ⋅ 10^−8^
4.	Cytoplasm	658	30.8	2.7 ⋅ 10^−8^
5.	Nucleoplasm	412	19.3	7.2 ⋅ 10^−7^
6.	Protein binding	1134	53.1	2.6 ⋅ 10^−6^
7.	Protein transport	112	5.2	5.5 ⋅ 10^−6^
8.	Nucleus	668	31.3	8.3 ⋅ 10^−5^
9.	Vesicle–mediated transport	39	1.8	1.9 ⋅ 10^−2^
10.	Cytoskeleton	177	8.3	8.6 ⋅ 10^−4^
11.	Rab GTPase binding	34	1.6	1.0 ⋅ 10^−2^
12.	Endocytosis	52	2.4	4.1 ⋅ 10^−3^
13.	Cytosol	450	21.1	6.0 ⋅ 10^−3^
14.	Guanine–nucleotide releasing factor	35	1.6	2.1 ⋅ 10^−3^
15.	Cell cycle	106	5.0	3.0 ⋅ 10^−3^
16.	Mitochondrion	162	7.6	4.1 ⋅ 10^−3^
17.	Transport	271	12.7	1.4 ⋅ 10^−2^
18.	Cell division	68	3.2	1.5 ⋅ 10^−2^
19.	Electron transport	24	1.1	1.4 ⋅ 10^−2^

DAVID functional annotation [48] was applied to the 2,136 RefSeq genes with a CpG dinucleotide at TSS position −1. Duplicated terms from different databases were deleted and the one with smallest p-value was retained. Shown are terms with adjusted p–value of *p* < 0.05 (Benjamini-Hochberg correction).

We then repeated the GO-term enrichment analysis at position −1/+1 for the remaining 15 dinucleotides and found only four other dinucleotides with several significant GO-term enrichments: CpA, GpA, TpA, and ApA, see S9 to S12 Tables. The genes harboring these three dinucleotides at position −1 were significantly associated with olfaction. This in fact suggests that olfactory genes often have an A at position −1. None of these dinucleotides provided a similarly high statistical enrichment as genes with a CpG dinucleotide at this position. It was reported before that olfactory receptor genes are associated with high AT content in their promoter region [49]. Nevertheless, we found only the three mentioned dinucleotides to be significantly associated with olfaction instead of other A-T dinucleotide combinations. The occurrence of different promoter compositions suggests that specific dinucleotides right at the transcription start site might be involved in transcriptional (and translational) regulation. This involves particularly CpG and CpA dinucleotides, which can be additionally epigenetically modified by DNA methylation. The observation that the dinucleotide present at position −1 is linked with the activity and expression level of a TSS [40] together with dinucleotide associated GO–terms indicates the involvement of specific dinucleotides right at the TSS in gene-group specific regulation. Furthermore, Liang *et al*. analyzed the usage of TSSs in different samples [50]. They categorized genes into genes that use the same TSS in different samples but are differentially expressed in these samples (denoted as class 1) and into genes that use alternative TSS in different samples (denoted as class 2). Interestingly, the term “acetylation” was found to be significantly enriched in both gene classes, whereas “phosphorylation” and “phosphoprotein” were only enriched in class 2 genes that utilize a unique TSS region in colon tissue [50]. Moreover, functional enrichment analysis of TSS data from time-course experiments in mouse dendritic cells resulted in a significant enrichment of the terms “phosphoprotein” and “acetylation” for class 1 genes, and “alternative splicing” (these are genes that regulate the alternative RNA splicing of other genes) and “phosphoprotein” for class 2 genes [50]. We suggest that the connection of “phosphoprotein” to “alternative splicing” reported in [50] may be of relevance for the interpretation of our findings. It was also reported that alternative promoters can arise from epigenetic changes such as DNA methylation [51]. In summary, these findings suggest that the detected signal at position −1 upstream of the TSS might also be associated with differential expression and alternative TSS or promoter usage.

#### CSS—Coding/translation start site

Since changes at a single position in close proximity to a start site can have an influence on translation initiation [16–19], we analyzed in detail the flanking region of canonical translation start sites and of alternative starts located in the 5’ UTR.

**Fig 5:**
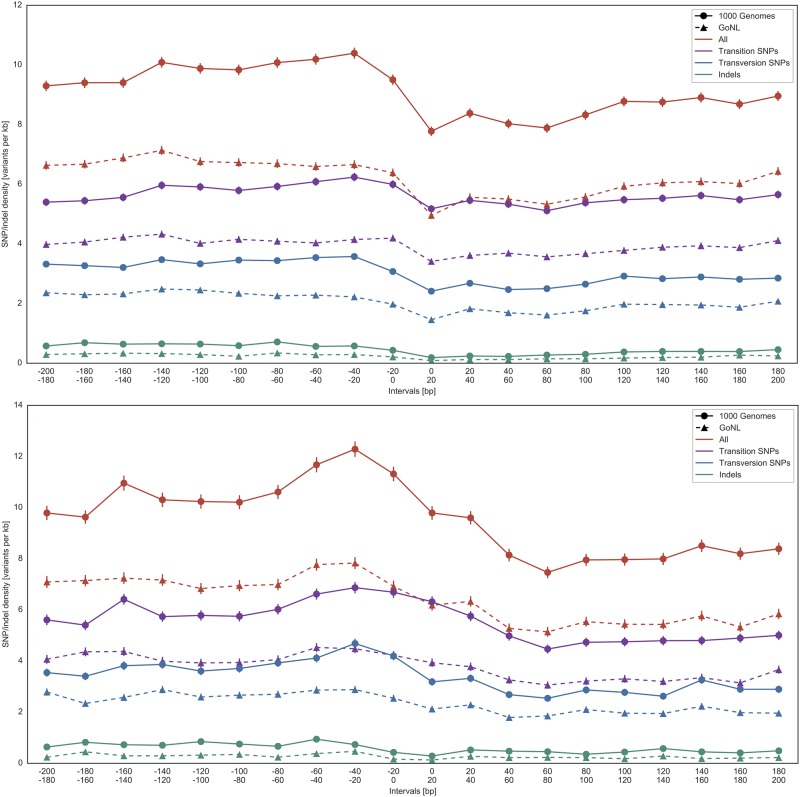
SNP distribution around the coding start site (CSS). Average SNP density in a range of ±200 bp and 20 bp windows around the CSS (1000G and GoNL data). Upper panel: annotated translation start sites of (RefSeq genes); Lower panel: alternative translation starts detected by ribosome profiling applied to HEK293 cells. The standard error of the mean is visualized for every datapoint.

First, we investigated the SNP and indel occurrence from −200 to +200 bp around translation start sites. Fig 5 shows that the SNP density decreases with the CSS for the annotated RefSeq start sites as well as for the alternative start sites located in the 5’ UTR that were identified by experimental ribosome profiling [21]. This depletion of SNPs in the coding region is most likely due to purifying selection. This effect is most prominent for indels, as this type of mutation has the potential to change the overall identity of proteins by shifts in the open reading frame. However, based on the overall small number of indels, we can only observe a small decrease of the indel density towards the CSS. A depletion of indels especially in protein coding regions was observed before and the observed Tajima’s D values are consistent with purifying selection, see Fig 3. SNP densities calculated based on 1000G and GoNL data behave similarly. The vertical shift between data from those two projects can be attributed to a more constrained population resulting in fewer SNPs.

Next, we focused on the start codon and the flanking region (−15 to +13) that has been shown to be crucial for translation initiation [16–19]. In total, the RefSeq dataset provided 16,604 canonical translation start sites (i.e. flanking regions), whereas the number of alternative start sites located in the 5’ UTR amounted to 7,373 [21]. SNPs from 1000G and GoNL were assigned to these sequence contexts to determine their position in the interval from −15 to +13 with respect to the respective start site. Table 1 summarizes the number of translational sequence contexts in the RefSeq (AUG-only) and HEK293 datasets (AUG and near-cognate) that harbor SNPs as well as the number of SNPs residing at those start sites. Table 1 reveals that, on average, there is about one SNP per start site flanking region. In the next step, we investigated the distribution of these SNPs along the defined sequence window from positions −15 to +13.

**Fig 6:**
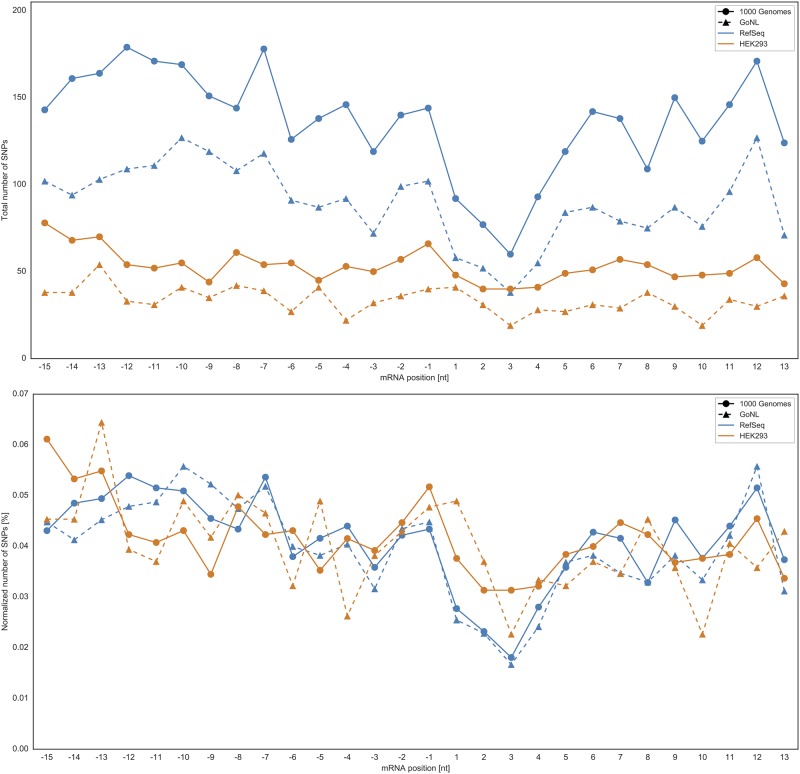
SNP distribution in the flanking region of the coding start site (CSS). SNP pattern in the flanking region (–15 to +13) of canonical and alternative starts. The applied permutation test provided the following p-values (curves from top to bottom): RefSeq+1000G: 0.0, RefSeq+GoNL: 0.0002, HEK293+1000G: 0.027, HEK293+GoNL: 0.244. With a significance threshold of $p<\frac{0.05}{4}=0.0125$, the drop in the total number of SNPs at the CSS is significant only for canonical start sites. Upper panel: total number of SNPs; Lower panel: the number of SNPs normalized by the number of sequence contexts with at least one SNP, compare with Table 1.

Fig 6 shows the total number of SNPs in the flanking region of annotated and alternative start sites. For convenience, we show the total number of SNPs (upper panel) as well as a normalization by the number of sequence contexts with at least one SNP (lower panel), compare with Table 1. We found that alternative start sites (AUG and near-cognate) located in the 5’ UTR showed similar conservation tendencies compared to annotated canonical start sites. As expected, the number of SNPs decreased remarkably with the start site reflecting the importance of the start codon. Moreover, the expected three periodicity of SNP densities [14, 15] is visible within the coding region of canonically translated proteins, indicating that the third codon base is generally less conserved compared to the other positions. To validate the statistical significance of this decrease in the total number of SNPs at the start site, we performed a permutation test. We found that the drop at canonical start sites (RefSeq genes) was highly significant irrespective of the mutation dataset (*p* < 0.01), see Fig 6. Considering alternative translation start sites in HEK293 cells, the decrease in the number of SNPs at the start codon (see Fig 6) is only moderate compared to the RefSeq starts and so are the p-values, which were found to be not significant.

A negative peak can also be observed at position −3, which was shown to be crucial for translation initiation [16, 17]. However, also this trend is more prominent for annotated start sites compared to alternative start sites (see Fig 6 (upper panel)). Comparing the normalized values in Fig 6 (lower panel), emphasizes our previous statement that canonical and alternative start sites show similar conservation patterns. Beside the start site itself and the prominent −3 position, several other positions also show negatives peaks, e.g. positions 8 or 10. Based on a simple permutation test, the drop at these positions is not statistically significant.

Thus, canonical translation start sites are well conserved to preserve normal cell behavior. Alternative initiation start sites seem to be less conserved compared to canonical start codons. This could be due to the location of the considered alternative start sites in the generally less conserved 5’ UTR as well as be related to the general usage of these alternative sites. Non-canonical start codons could be, for instance, used in specific cellular states, in specific cell types (here HEK293 cells) or as cellular stress response [22, 25]. Moreover, it is also possible that the dataset, based on the ribosome profiling technique, might contain some false positive start sites due to experimental and post-experimental (e.g. statistical evaluation) limitations [29].

## Conclusion

Analyzing SNPs and indels from the 1000G and the GoNL project revealed pronounced differences in the distribution of several variant types across genomic elements, such as promoters, 5’ UTRs, and coding exons. In general, SNP and indel frequencies found here are in accordance with earlier findings [13]–[15]. However, we discovered a remarkable amount of genes with a CpG dinucleotide right at the TSS (position −1) that coincided with an elevated number of SNPs at this position. Applying DAVID GO-term enrichment analysis, we found that most of these genes are significantly associated with the terms “Phosphoprotein”, “Alternative splicing” and “Protein binding”. One might speculate that a methylated CpG dinucleotide at this position functions as cellular signal for transcriptional regulation of specific gene groups. With respect to the translation start, our investigations showed that alternative start sites (AUG and near-cognate as well as in- and out-of-frame with the annotated start site) located in the human 5’ UTR exhibit a similar conservation tendency in their flanking region compared to annotated canonical AUG start sites. We found a pronounced decrease in the number of SNPs at the start site itself, but also at prominent position −3, which was experimentally shown to be crucial for translation initiation [16, 17]. In general, alternative starts are not as conserved as canonical start sites. Nevertheless, the similar conservation pattern found confirms the importance of alternative start codons (AUG and near-cognate), and their relevant contribution to the expansion of biological variety and complexity.

## Materials and methods

### Single nucleotide polymorphisms (SNPs) and indels

Information about annotated SNPs and indels in human genes was used from the 1000 Genomes Project (1000G, phase 3, using only the European super population EUR, 503 individuals) [9] and from the Genome of the Netherlands (GoNL) project (release 5) [10, 11]. Thereby, we used SNPs only from the parents, i.e. no data was pooled. Moreover, the calculated SNP density is a function of the cohort size because larger studies pick up lower allele frequencies. Data was provided in VCF file format. For the analyses, we kept autosomal SNPs with a minor allele frequency larger than zero (allele frequencies were calculated by the respective consortium, see [9–11]). These variants were assigned to four classes, namely transition SNPs, transversion SNPs, indels (insertions and deletions without length cutoff), and the union of all variants.

#### SNPs and indels in nine genomic elements

Human gene annotations were downloaded from the UCSC genome browser hg19 assembly (RefSeq genes). We removed genes coding for microRNAs and small nucleolar RNAs, genes with CDS start equal to the CDS end as well as genes located on chromosomes other than chromosome 1 to chromosome 22. Special care was taken of overlapping genes, where we distinguished between overlaps located inside other genes and staggered overlaps (genes overlap partially). Genes inside other genes were excluded. All genes with staggered overlap were collected and from each “bundle”, only one gene was selected to avoid overlapping genes. If a gene has more than one transcript variant, only the longest transcript was retained.

For a general overview on SNP frequencies in the human genome, nine basic genomic regions were derived based on the genomic information provided by the UCSC genome browser. The information needed to calculate the genomic coordinates of these regions for every gene was downloaded from UCSC genome browser and includes chromosome, strand, transcription start site (TSS), transcription end site (TES), CDS start (coding start site—CSS), CDS end (coding end site—CES), exon starts and exon ends. These regions comprise: intergenic region, CpG islands, promoter region, 5’ UTR, coding exons, 3’ UTR, all exons, introns, and intragenic region, see Fig 1. The regions were defined in the following way: every gene is located between two intergenic regions. The first one is defined as the interval between the TSS of the considered gene and the mid-upstream position between this TSS and the TES of the closest upstream gene. The second intergenic region is defined analogously according to the TSS of the closest downstream gene. The intragenic region of a gene is defined as the part between its TSS and its TES. The gene promoter was defined as the region from 2000 bp upstream to 1000 bp downstream of the TSS and thus overlaps with the intergenic region. 5’ UTRs are defined as the exonic segments between the TSS and the CSS while 3’ UTRs are defined analogously as the exonic regions between the CES and the TES. Exons are defined as the intervals between the exon start positions and exon end positions as given in the file retrieved from the UCSC genome browser. Introns are defined as the regions between the exonic gene parts. Besides these nine general regions, we also considered narrow sequence windows of ±200 bps around transcription and translation start sites as well as in direct vicinity (–15 to +13 bps) of the TSS and CSS.

Any calculations requiring interval arithmetic and sequence mapping were implemented using the BEDTools suite (version v2.26.0) [52], samtools (version 1.3.1) [53, 54] and/or bowtie (version 1.1.2) [55]. These operations include the assignment of SNPs and indels to their respective genes and genomic elements as well as the retrieval of genomic coordinates given a short nucleotide sequence and vice versa. SNP densities (number of SNPs per kb) were then calculated for the different variant types and the nine basic types of genomic elements. Note that the calculated SNP density is a function of the cohort size because larger studies pick up lower allele frequencies. The evaluation of the neutral evolution hypothesis was analyzed by the widely-used Tajima’s D statistic [31] for every genomic element. For this, we applied VCFtools (version v0.1.13) [56] with a bin size of 1 Mb to filtered VCF variant files that only contain variants found in the respective genomic regions. This means that these are concatenated variant files where regions of one type have been concatenated one after another. Tajima’s D aims at testing for the neutral mutation hypothesis by comparing two nucleotide diversity measures for genetic variation: the number of segregating sites and the average sequence diversity (number of nucleotide differences) [31]. Tajima’s D was only applied to the SNP data from 1000G because it provides publicly accessible genotype information. Therefore, the 1000G variations were split up into nine VCF files based on the genomic region they reside in. Tajima’s D was then computed separately for each of the nine VCF files using VCFtools and a bin size of 1 Mb. Genomic regions were then compared with each other using boxplots.

Two-tailed Wilcoxon rank sum tests together with Bonferroni correction were used for the statistical comparison of the different SNP types within the nine genomic elements. Thereby, we assume a p-value to be significant if *p* < 1.4 × 10^−3^ which is equal to $\frac{0.05}{\#tests}$ where $\#tests=\frac{9\times 8}{2}$.

### SNP and indel frequencies around the TSS and the CSS

Since the regions around transcription start sites (TSS) and translation/coding start sites (CSS) have direct effects on gene transcription and protein translation, we investigated these regions in more detail and at higher resolution with respect to their SNP and indel distribution. SNPs and indels can, for instance, influence the binding of transcription factors in the promoter region or the translation initiation of the ribosome scanning complex in the 5’ UTR. We therefore examined the average SNP density in a range of ±200 bp around the TSS and CSS, and subsequently focused on SNPs in direct vicinity (position −15 to +13) to transcription and translation start sites.

To analyze the significance of a reported peak in the TSS, we randomly shuffled the defined intervals using BEDTools shuffle, repeated our evaluation 1000 times and recorded the size of the highest peak. The distribution of the height of the highest peak was then compared with our reported peak.

As especially translation initiation was shown to be highly dependent on the start site flanking region [16–19], we analyzed annotated (RefSeq genes) as well as alternative start sites located in the 5’ UTR in detail. As alternative start codons we considered codons in the upstream 5’ UTR region of the annotated start codon that are either AUG or differ from AUG in one nucleotide position [20–22]. RefSeq genes were retrieved as described above while alternative start sites in human HEK293 cells were retrieved from experimental ribosome profiling data and used as annotated by the original authors [21]. To investigate the flanking region around translation start sites, we defined a sequence window from −15 to +13 relative to a start site that encompasses positions 1, 2 and 3, see Fig 2. Next, duplicated sequence contexts (for example from several transcript variants) and codons differing from AUG and near-cognate variants were removed. SNPs from 1000G and GoNL were then mapped to these sequence contexts. Indels were excluded from further analysis since the amount of indels located in the predefined sequence window from −15 to +13 was too small such that a profound significance analysis was not possible.

We conducted a permutation test to investigate whether the distribution of variations at the CSS (positions 1, 2, and 3) is statistically significant, i.e. we calculated the probability to detect a negative peak of a certain magnitude by random sampling. For this, sequence contexts were represented as binary strings, with 1 representing a SNP at a position, and 0 otherwise. First, for all mutated CSS sequence contexts detected by our analyses, in the following denoted as *M* for mutated sequence, we calculated the z-score:

$$\begin{array}{c} \phi_{M}=\frac{\mu_{M\{p\}}-C_{p}}{\sigma_{M\{p\}}} \end{array}$$

with the average number of SNPs over all positions *μ* (except positions at the CSS, i.e. *p* ∉ [1, 2, 3]), the average number *C*_*p*_ at positions *p* ∈ [1, 2, 3], and the respective standard deviation *σ*. We then randomly shuffled (*SH*) all binary sequence contexts (e.g. 00101 can be shuffled into 10100 or 11000 by switching positions randomly) and calculated analogously:

$$\begin{array}{c} \phi_{SH}=\frac{\mu_{SH\{p\}}-C_{p}}{\sigma_{SH\{p\}}} \end{array}$$

and used 10,000 repetitions to compute empirical p-values. We deem a p-value significant (Bonferroni corrected) if $p<\frac{0.05}{4}=0.0125$ (#*tests* = 4, when considering canonical and alternative start sites as well as 1000G and GoNL data).

## Supporting information

S1 Code Python scripts used for filtering, analysis, statistical testing and plotting. (ZIP)

S1 Fig SNP and indel densities for all genomic elements considering GoNL data. Shown are SNP and indel densities for all genomic elements considering the GoNL data. The horizontal line (−) represents the median value, the asterisk (⋆) denotes the mean value.
(PDF)

S2 Fig Lengths of the genomic elements (kb). Upper and lower panel show the same data but use different y-scales (0–200 kb and 0–10 kb). Each boxplot is labeled with the percentage of genes exhibiting this element [%], the median (⋆)[kb] and mean values (−) [kb]. 5’ UTRs are the shortest genomic elements with an median value of 180 bp (0.18 kb) whereas intergenic regions (median: ∼ 29 kb), introns (median: ∼ 24 kb) and the intragenic region (median: ∼ 25 kb) are the largest elements.
(PDF)

S3 Fig Average number of CpGs around the TSS of RefSeq genes. We considered a window from −5000 bp to +5000 bp around the TSS of RefSeq genes. Given is the absolute number of CpGs per interval of 500 bps length. The number of CpGs peaks at the TSS as was reported earlier [34].
(PDF)

S4 Fig Distribution of all 16 dinucleotides in the flanking region of the transcription start site (TSS). We considered all RefSeq genes that remained after filtering. Position 1 denotes the first intragenic nucleotide. A CpG dinucleotide at position −1 means that the C is located at position −1 while the G resides at position +1.
(PDF)

S5 Fig Number of SNPs at individual dinucleotides in the flanking region of transcription start sites (TSS) considering 1000G data. Shown is the number of SNPs at individual dinucleotides in the flanking region of the TSS considering the 1000G data. SNPs were analyzed at individual dinucleotides in the flanking region of the TSS. Position 1 denotes the first intragenic nucleotide.
(PDF)

S6 Fig Number of SNPs at individual dinucleotides in the flanking region of transcription start sites (TSS) considering GoNL data. SNPs were analyzed at individual dinucleotides in the flanking region of the TSS. Depicted is the number of SNPs at individual dinucleotides in the flanking region of the TSS considering the GoNL data. Position 1 denotes the first intragenic nucleotide.
(PDF)

S1 Table SNP and indel densities for all genomic regions concerning the 1000G data. SNP and indel densities for all genomic regions concerning the 1000G data. Shown are mean, median and standard deviation.
(PDF)

S2 Table SNP and indel densities for all genomic regions concerning the GoNL data. SNP and indel densities for all genomic regions concerning the GoNL data. Shown are mean, median and standard deviation.
(PDF)

S3 TableResults of the statistical tests for the 1000G data comparing variant types within all gene regions.Two-tailed Wilcoxon rank sum tests together with Bonferroni correction were used for the statistical comparison of the different SNP types within the nine genomic elements. Thereby, we assume a p-value to be significant if *p* < 1.4 × 10^−3^ which is equal to $\frac{0.05}{\#tests}$ where $\#tests=\frac{9\times 8}{2}$. Note that due to numerical reasons, very small p–values (< 10^−310^) are represented as 0.0 in python programming language.(PDF)

S4 TableResults of the statistical tests for the 1000G data comparing gene regions for all variant types.Two-tailed Wilcoxon rank sum tests together with Bonferroni correction were used for the statistical comparison of the different SNP types within the nine genomic elements. Thereby, we assume a p-value to be significant if *p* < 1.4 × 10^−3^ which is equal to $\frac{0.05}{\#tests}$ where $\#tests=\frac{9\times 8}{2}$. Note that due to numerical reasons, very small p–values (< 10^−310^) are represented as 0.0 in python programming language.(PDF)

S5 TableResults of the statistical tests for the GoNL data comparing variant types within all gene regions.Two-tailed Wilcoxon rank sum tests together with Bonferroni correction were used for the statistical comparison of the different SNP types within the nine genomic elements. Thereby, we assume a p-value to be significant if *p* < 1.4 × 10^−3^ which is equal to $\frac{0.05}{\#tests}$ where $\#tests=\frac{9\times 8}{2}$. Note that due to numerical reasons, very small p–values (< 10^−310^) are represented as 0.0 in python programming language.(PDF)

S6 TableResults of the statistical tests for the GoNL data comparing gene regions for all variant types.Two-tailed Wilcoxon rank sum tests together with Bonferroni correction were used for the statistical comparison of the different SNP types within the nine genomic elements. Thereby, we assume a p-value to be significant if *p* < 1.4 × 10^−3^ which is equal to $\frac{0.05}{\#tests}$ where $\#tests=\frac{9\times 8}{2}$. Note that due to numerical reasons, very small p–values (< 10^−310^) are represented as 0.0 in python programming language.(PDF)

S7 Table Top100 genes with largest SNP density in coding exons. Shown are the Top100 genes with the largest SNP densities from the European cohort of the 1000G set by sorting with respect to coding exons.
(PDF)

S8 Table Top100 genes with largest SNP density in promoter regions. Shown are the Top100 genes with the largest SNP densities from the European cohort of the 1000G set by sorting with respect to promoter regions.
(PDF)

S9 Table Results of DAVID functional annotation considering Ap* dinucleotides. Results of DAVID functional annotation [48] for all genes that contain such dinucleotides at TSS position −1. Duplicated terms from different databases were deleted and the one with smallest p–value was retained. Shown are terms with corrected p–value of *p* < 0.05 (Benjamini correction). If no significant GO term enrichment was found for a dinucleotide gene subset, only the first two terms are displayed for convenience. The number of genes (RefSeq identifiers accepted by DAVID tool) of every subgroup is given in brackets.
(PDF)

S10 Table Results of DAVID functional annotation considering Cp* dinucleotides. Results of DAVID functional annotation [48] for all genes that contain such dinucleotides at TSS position −1. Duplicated terms from different databases were deleted and the one with smallest p–value was retained. Shown are terms with corrected p–value of *p* < 0.05 (Benjamini correction). If no significant GO term enrichment was found for a dinucleotide gene subset, only the first two terms are displayed for convenience. The number of genes (RefSeq identifiers accepted by DAVID tool) of every subgroup is given in brackets.
(PDF)

S11 Table Results of DAVID functional annotation considering Gp* dinucleotides. Results of DAVID functional annotation [48] for all genes that contain such dinucleotides at TSS position −1. Duplicated terms from different databases were deleted and the one with smallest p–value was retained. Shown are terms with corrected p–value of *p* < 0.05 (Benjamini correction). If no significant GO term enrichment was found for a dinucleotide gene subset, only the first two terms are displayed for convenience. The number of genes (RefSeq identifiers accepted by DAVID tool) of every subgroup is given in brackets.
(PDF)

S12 Table Results of DAVID functional annotation considering Tp* dinucleotides. Results of DAVID functional annotation [48] for all genes that contain such dinucleotides at TSS position −1. Duplicated terms from different databases were deleted and the one with smallest p–value was retained. Shown are terms with corrected p–value of *p* < 0.05 (Benjamini correction). If no significant GO term enrichment was found for a dinucleotide gene subset, only the first two terms are displayed for convenience. The number of genes (RefSeq identifiers accepted by DAVID tool) of every subgroup is given in brackets.
(PDF)

## References

[1] Barreiro LB, Laval G, Quach H, Patin E, Quintana-Murci L. Natural selection has driven population differentiation in modern humans. Nat Genet. 2008;40(3):340–345. 10.1038/ng.78 18246066

[2] Cheung VG, Spielman RS. Genetics of human gene expression: mapping DNA variants that influence gene expression. Nat Rev Genet. 2009;10(9):595–604. 10.1038/nrg2630 19636342 PMC2989458

[3] Syvänen AC. Accessing genetic variation: genotyping single nucleotide polymorphisms. Nat Rev Genet. 2001;2(12):930–942. 10.1038/35103535 11733746

[4] H E, F GM. Fidelity Mechanisms in DNA Replication. Annu Rev Biochem. 1991;60:477–511. 10.1146/annurev.bi.60.070191.002401 1883202

[5] Cooke MS, Evans MD, Dizdaroglu M, Lunec J. Oxidative DNA damage: mechanisms, mutation, and disease. FASEB J. 2003;17(10):1195–1214. 10.1096/fj.02-0752rev 12832285

[6] Branzei D, Foiani M. Regulation of DNA repair throughout the cell cycle. Nat Rev Mol Cell Biol. 2008;9(4):297–308. 10.1038/nrm2351 18285803

[7] Nielsen R, Hubisz MJ, Hellmann I, Torgerson D, Andres AM, Albrechtsen A, et al Darwinian and demographic forces affecting human protein coding genes. Genome Res. 2009;19(5):838–849. 10.1101/gr.088336.108 19279335 PMC2675972

[8] Jiang C, Zhao Z. Directionality of point mutation and 5-methylcytosine deamination rates in the chimpanzee genome. BMC Genomics. 2006;7:316 10.1186/1471-2164-7-316 17166280 PMC1764022

[9] The 1000 Genomes Project Consortium. A global reference for human genetic variation. Nature. 2015;526(7571):68–74. 10.1038/nature15393 26432245 PMC4750478

[10] The Genome of the Netherlands Consortium. Whole-genome sequence variation, population structure and demographic history of the Dutch population. Nat Genet. 2014;46(8):818–825. 10.1038/ng.3021 24974849

[11] Francioli LC, Polak PP, Koren A, Menelaou A, Chun S, Renkens I, et al Genome-wide patterns and properties of de novo mutations in humans. Nat Genet. 2015;47(7):822–826. 10.1038/ng.3292 25985141 PMC4485564

[12] Kloosterman WP, Francioli LC, Hormozdiari F, Marschall T, Hehir-Kwa JY, Abdellaoui A, et al Origin, frequency and functional impact of de novo structural changes in the human genome. Genome Research. 2015;25:792–801. 25883321 10.1101/gr.185041.114 PMC4448676

[13] Telenti A, Pierce LC, Biggs WH, di Iulio J, Wong EH, Fabani MM, et al Deep sequencing of 10,000 human genomes. Proc Natl Acad Sci USA. 2016;113(42):11901–11906. 10.1073/pnas.1613365113 27702888 PMC5081584

[14] Castle JC. SNPs occur in regions with less genomic sequence conservation. PLoS ONE. 2011;6(6):e20660 10.1371/journal.pone.0020660 21674007 PMC3108954

[15] Tatarinova TV, Chekalin E, Nikolsky Y, Bruskin S, Chebotarov D, McNally KL, et al Nucleotide diversity analysis highlights functionally important genomic regions. Sci Rep. 2016;6:35730 10.1038/srep35730 27774999 PMC5075931

[16] Kozak M. Compilation and analysis of sequences upstream from the translational start site in eukaryotic mRNAs. Nucleic Acids Res. 1984;12:857–872. 10.1093/nar/12.2.857 6694911 PMC318541

[17] Kozak M. Point mutations define a sequence flanking the AUG initiator codon that modulates translation by eukaryotic ribosomes. Cell. 1986;44:283–292. 10.1016/0092-8674(86)90762-2 3943125

[18] Noderer WL, Flockhart RJ, Bhaduri A, Diaz de Arce AJ, Zhang J, Khavari PA, et al Quantitative analysis of mammalian translation initiation sites by FACS-seq. Mol Syst Biol. 2014;10:748 10.15252/msb.20145136 25170020 PMC4299517

[19] He Y, Maier K, Leppert J, Hausser I, Schwieger-Briel A, Weibel L, et al Monoallelic Mutations in the Translation Initiation Codon of KLHL24 Cause Skin Fragility. Am J Hum Genet. 2016;99(6):1395–1404. 10.1016/j.ajhg.2016.11.005 27889062 PMC5142111

[20] Ivanov IP, Firth AE, Michel AM, Atkins JF, Baranov PV. Identification of evolutionarily conserved non-AUG-initiated N-terminal extensions in human coding sequences. Nucleic Acids Res. 2011;39:4220–4234. 10.1093/nar/gkr007 21266472 PMC3105428

[21] Lee S, Liu B, Lee S, Huang SX, Shen B, Qian SB. Global mapping of translation initiation sites in mammalian cells at single-nucleotide resolution. Proc Natl Acad Sci USA. 2012;109:E2424–2432. 10.1073/pnas.1207846109 22927429 PMC3443142

[22] de Klerk E, ’t Hoen PA. Alternative mRNA transcription, processing, and translation: insights from RNA sequencing. Trends Genet. 2015;31(3):128–139. 10.1016/j.tig.2015.01.001 25648499

[23] Ingolia NT. Ribosome profiling: new views of translation, from single codons to genome scale. Nat Rev Genet. 2014;15:205–213. 10.1038/nrg3645 24468696

[24] Hann SR, Dixit M, Sears RC, Sealy L. The alternatively initiated c-Myc proteins differentially regulate transcription through a noncanonical DNA-binding site. Genes Dev. 1994;8:2441–2452. 10.1101/gad.8.20.2441 7958908

[25] Vagner S, Touriol C, Galy B, Audigier S, Gensac MC, Amalric F, et al Translation of CUG- but not AUG-initiated forms of human fibroblast growth factor 2 is activated in transformed and stressed cells. J Cell Biol. 1996;135:1391–1402. 10.1083/jcb.135.5.1391 8947560 PMC2121090

[26] Touriol C, Bornes S, Bonnal S, Audigier S, Prats H, Prats AC, et al Generation of protein isoform diversity by alternative initiation of translation at non-AUG codons. Biol Cell. 2003;95:169–178. 10.1016/S0248-4900(03)00033-9 12867081

[27] Ingolia NT, Lareau LF, Weissman JS. Ribosome profiling of mouse embryonic stem cells reveals the complexity and dynamics of mammalian proteomes. Cell. 2011;147(4):789–802. 10.1016/j.cell.2011.10.002 22056041 PMC3225288

[28] Ingolia NT, Ghaemmaghami S, Newman JR, Weissman JS. Genome-wide analysis in vivo of translation with nucleotide resolution using ribosome profiling. Science. 2009;324:218–223. 10.1126/science.1168978 19213877 PMC2746483

[29] Brar GA, Weissman JS. Ribosome profiling reveals the what, when, where and how of protein synthesis. Nat Rev Mol Cell Biol. 2015;16(11):651–664. 10.1038/nrm4069 26465719 PMC5522010

[30] Reuter K, Biehl A, Koch L, Helms V. PreTIS: A Tool to Predict Non-canonical 5’ UTR Translational Initiation Sites in Human and Mouse. PLoS Comput Biol. 2016;12(10):e1005170 10.1371/journal.pcbi.1005170 27768687 PMC5074520

[31] Tajima F. Statistical method for testing the neutral mutation hypothesis by DNA polymorphism. Genetics. 1989;123(3):585–595. 2513255 10.1093/genetics/123.3.585 PMC1203831

[32] Taylor MS, Kai C, Kawai J, Carninci P, Hayashizaki Y, Semple CA. Heterotachy in mammalian promoter evolution. PLoS Genet. 2006;2(4):e30 10.1371/journal.pgen.0020030 16683025 PMC1449885

[33] Clark TG, Andrew T, Cooper GM, Margulies EH, Mullikin JC, Balding DJ. Functional constraint and small insertions and deletions in the ENCODE regions of the human genome. Genome Biol. 2007;8(9):R180 10.1186/gb-2007-8-9-r180 17784950 PMC2375018

[34] Saxonov S, Berg P, Brutlag DL. A genome-wide analysis of CpG dinucleotides in the human genome distinguishes two distinct classes of promoters. Proc Natl Acad Sci USA. 2006;103(5):1412–1417. 10.1073/pnas.0510310103 16432200 PMC1345710

[35] Norman PJ, Norberg SJ, Guethlein LA, Nemat-Gorgani N, Royce T, Wroblewski EE, et al Sequences of 95 human MHC haplotypes reveal extreme coding variation in genes other than highly polymorphic HLA class I and II. Genome Res. 2017;27(5):813–823. 10.1101/gr.213538.116 28360230 PMC5411776

[36] Higasa K, Hayashi K. Periodicity of SNP distribution around transcription start sites. BMC Genomics. 2006;7:66 10.1186/1471-2164-7-66 16579865 PMC1448210

[37] Sabarinathan R, Mularoni L, Deu-Pons J, Gonzalez-Perez A, Lopez-Bigas N. Nucleotide excision repair is impaired by binding of transcription factors to DNA. Nature. 2016;532(7598):264–267. 10.1038/nature17661 27075101

[38] Marino-Ramirez L, Spouge JL, Kanga GC, Landsman D. Statistical analysis of over-represented words in human promoter sequences. Nucleic Acids Res. 2004;32(3):949–958. 10.1093/nar/gkh246 14963262 PMC373387

[39] Rahbari R, Wuster A, Lindsay SJ, Hardwick RJ, Alexandrov LB, Turki SA, et al Timing, rates and spectra of human germline mutation. Nat Genet. 2016;48(2):126–133. 10.1038/ng.3469 26656846 PMC4731925

[40] Carninci P, Sandelin A, Lenhard B, Katayama S, Shimokawa K, Ponjavic J, et al Genome-wide analysis of mammalian promoter architecture and evolution. Nat Genet. 2006;38(6):626–635. 10.1038/ng1789 16645617

[41] Frith MC, Valen E, Krogh A, Hayashizaki Y, Carninci P, Sandelin A. A code for transcription initiation in mammalian genomes. Genome Res. 2008;18(1):1–12. 10.1101/gr.6831208 18032727 PMC2134772

[42] Mugal CF, Arndt PF, Holm L, Ellegren H. Evolutionary consequences of DNA methylation on the GC content in vertebrate genomes. G3 (Bethesda). 2015;5(3):441–447. 10.1534/g3.114.015545 25591920 PMC4349097

[43] Schmutte C, Yang AS, Beart RW, Jones PA. Base excision repair of U:G mismatches at a mutational hotspot in the p53 gene is more efficient than base excision repair of T:G mismatches in extracts of human colon tumors. Cancer Res. 1995;55(17):3742–3746. 7641186

[44] Bird A, Taggart M, Frommer M, Miller OJ, Macleod D. A fraction of the mouse genome that is derived from islands of nonmethylated, CpG-rich DNA. Cell. 1985;40(1):91–99. 10.1016/0092-8674(85)90312-5 2981636

[45] Patil V, Ward RL, Hesson LB. The evidence for functional non-CpG methylation in mammalian cells. Epigenetics. 2014;9(6):823–828. 10.4161/epi.28741 24717538 PMC4065179

[46] Pinney SE. Mammalian Non-CpG Methylation: Stem Cells and Beyond. Biology (Basel). 2014;3(4):739–751. 25393317 10.3390/biology3040739 PMC4280509

[47] Lister R, Pelizzola M, Dowen RH, Hawkins RD, Hon G, Tonti-Filippini J, et al Human DNA methylomes at base resolution show widespread epigenomic differences. Nature. 2009;462(7271):315–322. 10.1038/nature08514 19829295 PMC2857523

[48] Huang da W, Sherman BT, Lempicki RA. Systematic and integrative analysis of large gene lists using DAVID bioinformatics resources. Nat Protoc. 2009;4(1):44–57. 10.1038/nprot.2008.211 19131956

[49] Clowney EJ, Magklara A, Colquitt BM, Pathak N, Lane RP, Lomvardas S. High-throughput mapping of the promoters of the mouse olfactory receptor genes reveals a new type of mammalian promoter and provides insight into olfactory receptor gene regulation. Genome Res. 2011;21(8):1249–1259. 10.1101/gr.120162.110 21705439 PMC3149492

[50] Liang KC, Suzuki Y, Kumagai Y, Nakai K. Analysis of changes in transcription start site distribution by a classification approach. Gene. 2014;537(1):29–40. 10.1016/j.gene.2013.12.038 24389500

[51] Hatchwell E, Greally JM. The potential role of epigenomic dysregulation in complex human disease. Trends Genet. 2007;23(11):588–595. 10.1016/j.tig.2007.08.010 17953999

[52] Quinlan AR, Hall IM. BEDTools: a flexible suite of utilities for comparing genomic features. Bioinformatics. 2010;26(6):841–842. 10.1093/bioinformatics/btq033 20110278 PMC2832824

[53] Li H, Handsaker B, Wysoker A, Fennell T, Ruan J, Homer N, et al The Sequence Alignment/Map format and SAMtools. Bioinformatics. 2009;25(16):2078–2079. 10.1093/bioinformatics/btp352 19505943 PMC2723002

[54] Li H. A statistical framework for SNP calling, mutation discovery, association mapping and population genetical parameter estimation from sequencing data. Bioinformatics. 2011;27(21):2987–2993. 10.1093/bioinformatics/btr509 21903627 PMC3198575

[55] Langmead B, Trapnell C, Pop M, Salzberg SL. Ultrafast and memory-efficient alignment of short DNA sequences to the human genome. Genome Biol. 2009;10(3):R25 10.1186/gb-2009-10-3-r25 19261174 PMC2690996

[56] Danecek P, Auton A, Abecasis G, Albers CA, Banks E, DePristo MA, et al The variant call format and VCFtools. Bioinformatics. 2011;27(15):2156–2158. 10.1093/bioinformatics/btr330 21653522 PMC3137218

