# Supplementary figures and images for: SNP and indel frequencies at transcription start sites and at canonical and alternative translation initiation sites in the human genome
Source: PLoS One. 2019 Apr 12;14(4):e0214816. doi: 10.1371/journal.pone.0214816 (PMC6461226; doi:10.1371/journal.pone.0214816)

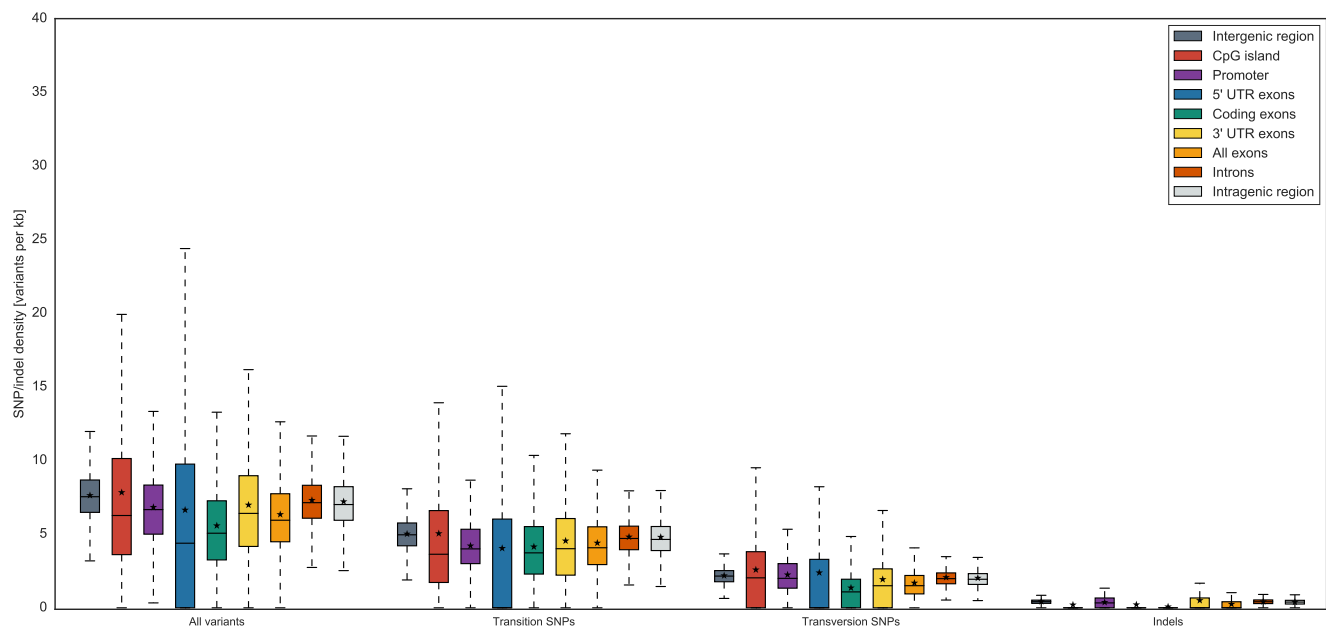

**S1 Fig.**

Supplement: S1 Fig — Shown are SNP and indel densities for all genomic elements considering the GoNL data. The horizontal line (−) represents the median value, the asterisk (⋆) denotes the mean value. (PDF) [file pone.0214816.s002.pdf]

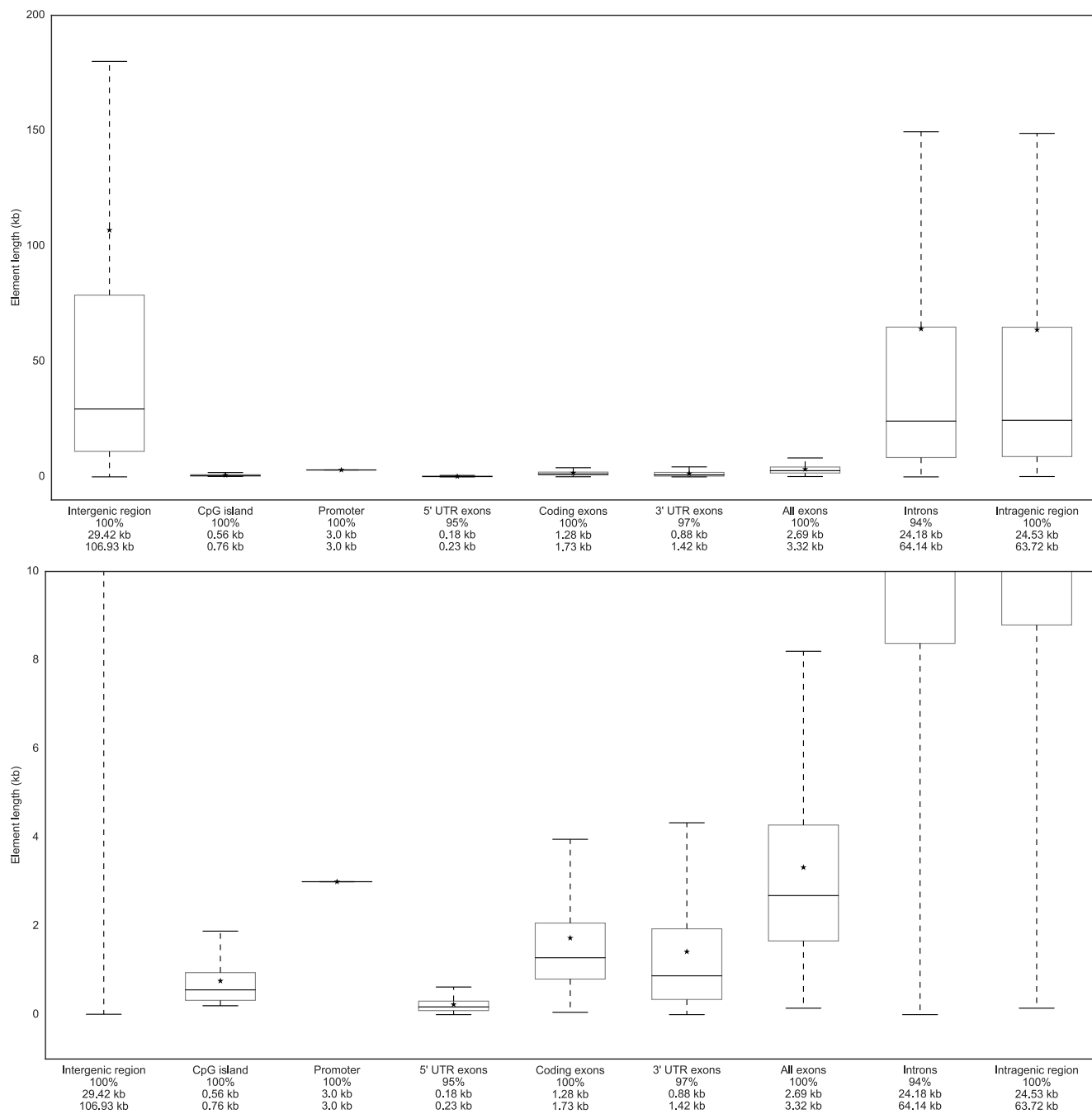

**S2 Fig.**

Supplement: S2 Fig — Upper and lower panel show the same data but use different y-scales (0–200 kb and 0–10 kb). Each boxplot is labeled with the percentage of genes exhibiting this element [%], the median (⋆)[kb] and mean values (−) [kb]. 5’ UTRs are the shortest genomic elements with an median value of 180 bp (0.18 kb) whereas intergenic regions (median: ∼ 29 kb), introns (median: ∼ 24 kb) and the intragenic region (median: ∼ 25 kb) are the largest elements. (PDF) [file pone.0214816.s003.pdf]

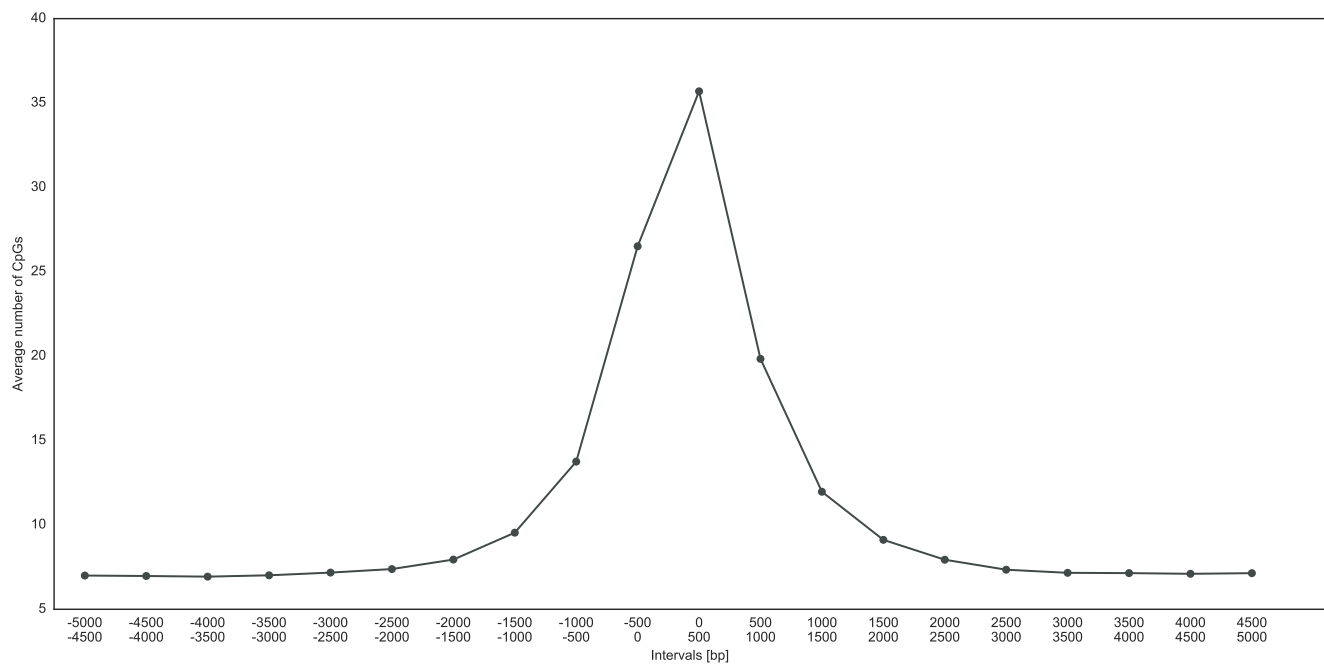

**S3 Fig.**

Supplement: S3 Fig — We considered a window from −5000 bp to +5000 bp around the TSS of RefSeq genes. Given is the absolute number of CpGs per interval of 500 bps length. The number of CpGs peaks at the TSS as was reported earlier [34]. (PDF) [file pone.0214816.s004.pdf]

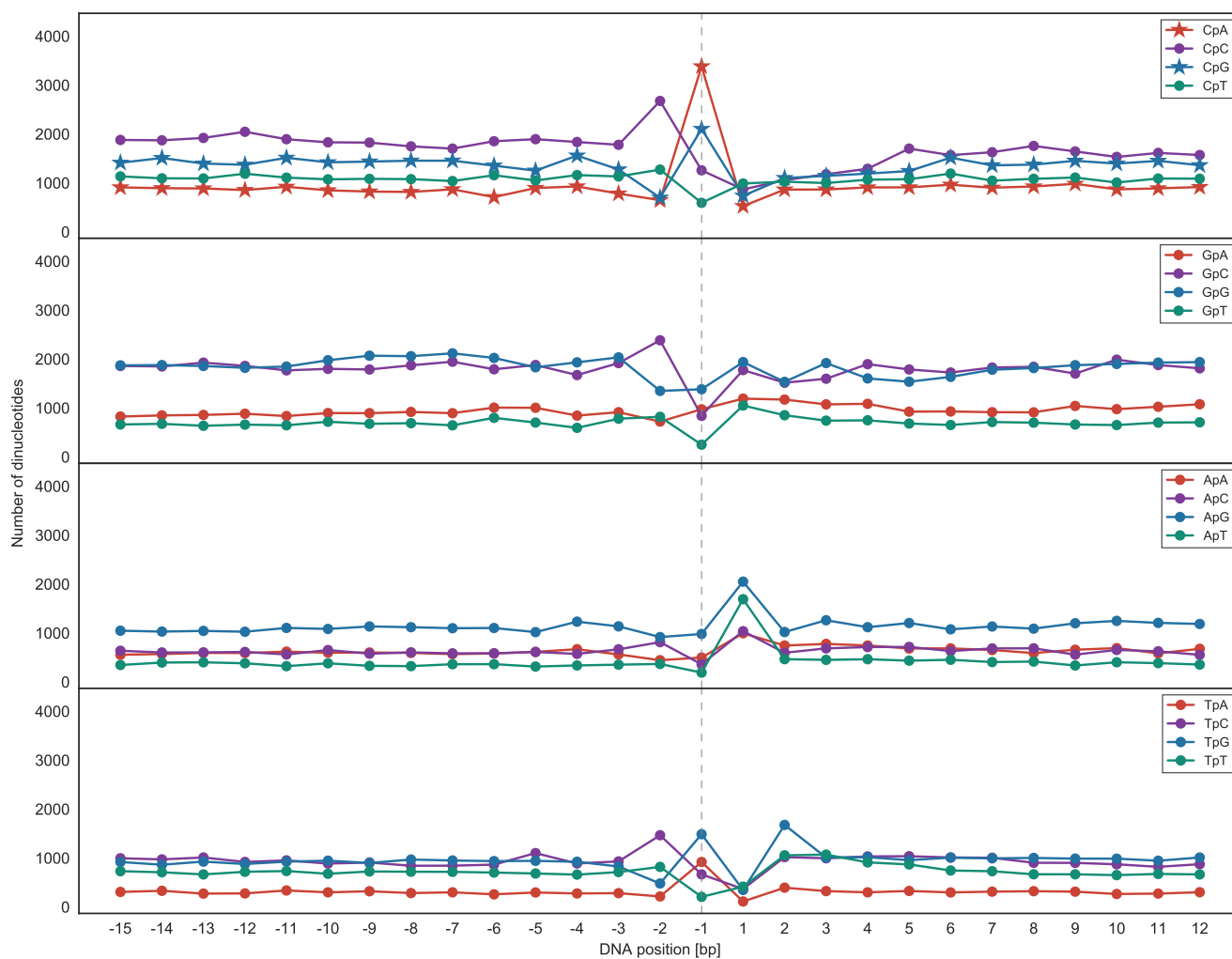

S4 Fig.

Supplement: S4 Fig — We considered all RefSeq genes that remained after filtering. Position 1 denotes the first intragenic nucleotide. A CpG dinucleotide at position −1 means that the C is located at position −1 while the G resides at position +1. (PDF) [file pone.0214816.s005.pdf]

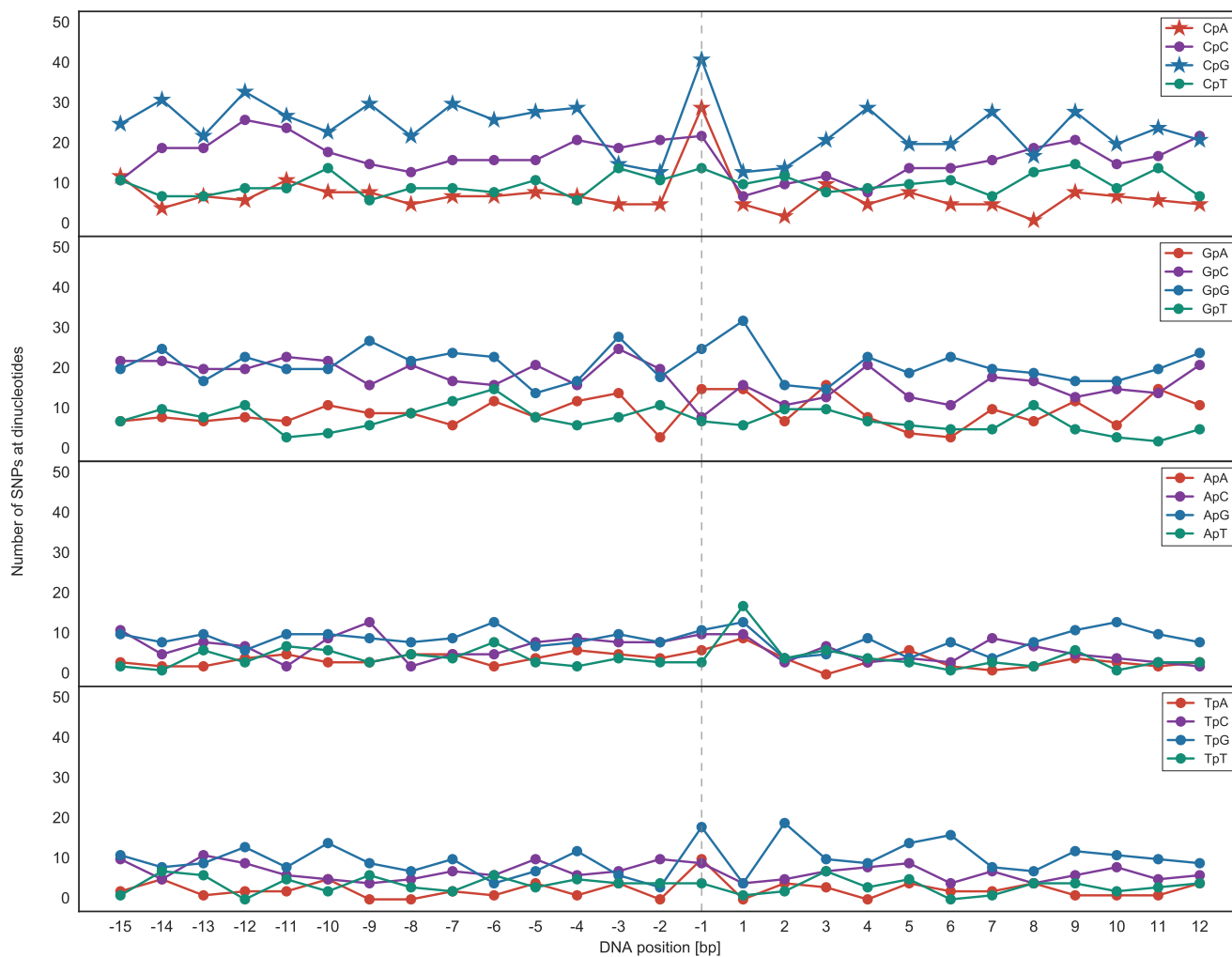

S5 Fig.

Supplement: S5 Fig — Shown is the number of SNPs at individual dinucleotides in the flanking region of the TSS considering the 1000G data. SNPs were analyzed at individual dinucleotides in the flanking region of the TSS. Position 1 denotes the first intragenic nucleotide. (PDF) [file pone.0214816.s006.pdf]

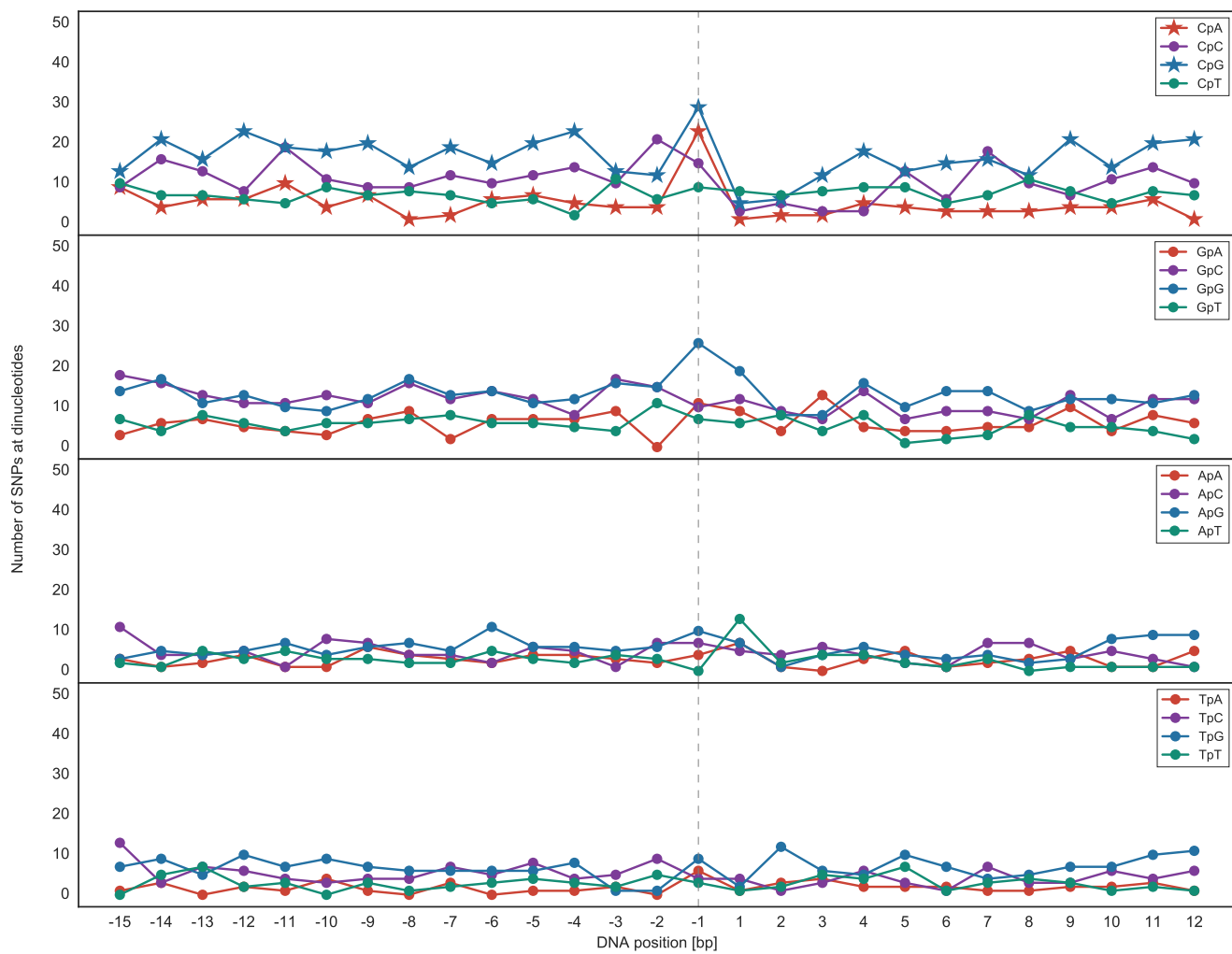

S6 Fig.

Supplement: S6 Fig — SNPs were analyzed at individual dinucleotides in the flanking region of the TSS. Depicted is the number of SNPs at individual dinucleotides in the flanking region of the TSS considering the GoNL data. Position 1 denotes the first intragenic nucleotide. (PDF) [file pone.0214816.s007.pdf]
