# Supplementary material for: SNP and indel frequencies at transcription start sites and at canonical and alternative translation initiation sites in the human genome
Source: PLoS One. 2019 Apr 12;14(4):e0214816. doi: 10.1371/journal.pone.0214816 (PMC6461226; doi:10.1371/journal.pone.0214816)
Supplement: S1 Table — SNP and indel densities for all genomic regions concerning the 1000G data. Shown are mean, median and standard deviation. (PDF) [file pone.0214816.s008.pdf]

S1 Table

**Intergenic region**			
	median value	mean value	standard deviation
All variants	8.8	8.99	2.53
Transition SNPs	5.61	5.72	1.74
Transversion SNPs	2.52	2.59	0.98
Indels	0.64	0.68	0.41
**CpG island**			
	median value	mean value	standard deviation
All variants	9.46	10.91	7.51
Transition SNPs	5.35	6.91	6.1
Transversion SNPs	3.07	3.49	3.24
Indels	0.0	0.51	1.1
**Promoter**			
	median value	mean value	standard deviation
All variants	8.33	8.67	3.22
Transition SNPs	5.0	5.19	2.22
Transversion SNPs	2.67	2.84	1.34
Indels	0.67	0.64	0.55
**5’ UTR exons**			
	median value	mean value	standard deviation
All variants	8.06	10.23	11.55
Transition SNPs	4.0	6.04	8.53
Transversion SNPs	0.0	3.68	6.71
Indels	0.0	0.51	2.13
**Coding exons**			
	median value	mean value	standard deviation
All variants	6.83	7.52	4.44
Transition SNPs	4.98	5.56	3.49
Transversion SNPs	1.56	1.87	1.77
Indels	0.0	0.09	0.36
**3’ UTR exons**			
	median value	mean value	standard deviation
All variants	7.61	8.46	6.52
Transition SNPs	4.73	5.42	5.05
Transversion SNPs	1.9	2.39	3.08
Indels	0.0	0.66	1.37
**All exons**			
	median value	mean value	standard deviation
All variants	7.59	8.18	3.72
Transition SNPs	5.15	5.63	2.84
Transversion SNPs	1.98	2.22	1.45
Indels	0.18	0.33	0.46
**Introns**			
	median value	mean value	standard deviation
All variants	8.39	8.74	3.05
Transition SNPs	5.37	5.62	2.16
Transversion SNPs	2.34	2.48	1.11
Indels	0.61	0.64	0.39
**Intragenic region**			
	median value	mean value	standard deviation
All variants	8.36	8.77	3.04
Transition SNPs	5.39	5.71	2.16
Transversion SNPs	2.32	2.48	1.09
Indels	0.57	0.58	0.34
