# Supplementary material for: SNP and indel frequencies at transcription start sites and at canonical and alternative translation initiation sites in the human genome
Source: PLoS One. 2019 Apr 12;14(4):e0214816. doi: 10.1371/journal.pone.0214816 (PMC6461226; doi:10.1371/journal.pone.0214816)
Supplement: S2 Table — SNP and indel densities for all genomic regions concerning the GoNL data. Shown are mean, median and standard deviation. (PDF) [file pone.0214816.s009.pdf]

S2 Table

**Intergenic region**			
	median value	mean value	standard deviation
All variants	7.54	7.63	2.21
Transition SNPs	4.96	5.02	1.55
Transversion SNPs	2.15	2.19	0.86
Indels	0.42	0.43	0.27
**CpG island**			
	median value	mean value	standard deviation
All variants	6.27	7.83	6.79
Transition SNPs	3.64	5.05	5.42
Transversion SNPs	2.03	2.58	2.95
Indels	0.0	0.2	0.7
**Promoter**			
	median value	mean value	standard deviation
All variants	6.67	6.83	2.77
Transition SNPs	4.0	4.21	1.98
Transversion SNPs	2.0	2.25	1.15
Indels	0.33	0.37	0.39
**5' UTR exons**			
	median value	mean value	standard deviation
All variants	4.39	6.64	9.23
Transition SNPs	0.0	4.04	7.04
Transversion SNPs	0.0	2.38	5.19
Indels	0.0	0.22	1.5
**Coding exons**			
	median value	mean value	standard deviation
All variants	5.06	5.58	3.53
Transition SNPs	3.73	4.15	2.82
Transversion SNPs	1.08	1.36	1.44
Indels	0.0	0.07	0.32
**3' UTR exons**			
	median value	mean value	standard deviation
All variants	6.41	6.98	5.36
Transition SNPs	4.01	4.55	4.22
Transversion SNPs	1.5	1.93	2.68
Indels	0.0	0.5	1.11
**All exons**			
	median value	mean value	standard deviation
All variants	5.95	6.34	2.97
Transition SNPs	4.08	4.4	2.29
Transversion SNPs	1.5	1.69	1.19
Indels	0.0	0.25	0.38
**Introns**			
	median value	mean value	standard deviation
All variants	7.14	7.31	2.39
Transition SNPs	4.71	4.82	1.69
Transversion SNPs	1.98	2.07	0.91
Indels	0.41	0.42	0.28
**Intragenic region**			
	median value	mean value	standard deviation
All variants	7.01	7.21	2.42
Transition SNPs	4.64	4.8	1.75
Transversion SNPs	1.94	2.02	0.87
Indels	0.39	0.39	0.25
