# Supplementary material for: SNP and indel frequencies at transcription start sites and at canonical and alternative translation initiation sites in the human genome
Source: PLoS One. 2019 Apr 12;14(4):e0214816. doi: 10.1371/journal.pone.0214816 (PMC6461226; doi:10.1371/journal.pone.0214816)
Supplement: S4 Table — Two-tailed Wilcoxon rank sum tests together with Bonferroni correction were used for the statistical comparison of the different SNP types within the nine genomic elements. Thereby, we assume a p-value to be significant if p < 1.4 × 10^−3^ which is equal to 0.05#tests where #tests=9×82. Note that due to numerical reasons, very small p–values (< 10−310) are represented as 0.0 in python programming language. (PDF) [file pone.0214816.s011.pdf]

S4 Table

All variants

	Intergenic region	CpG island	Promoter	5’ UTR exons	Coding exons	3’ UTR exons	All exons	Introns	Intragenic region
Intergenic region	1.00	2.07×10^{-64}	3.88×10^{-75}	1.80×10^{-32}	0.00	1.59×10^{-218}	0.00	1.46×10^{-55}	4.84×10^{-62}
CpG island	2.07×10^{-64}	1.00	7.07×10^{-159}	9.13×10^{-133}	0.00	0.00	0.00	1.78×10^{-129}	1.66×10^{-132}
Promoter	3.88×10^{-75}	7.07×10^{-159}	1.00	4.12×10^{-10}	0.00	1.46×10^{-76}	3.79×10^{-100}	1.12×10^{-07}	9.95×10^{-07}
5’ UTR exons	1.80×10^{-32}	9.13×10^{-133}	4.12×10^{-10}	1.00	1.86×10^{-27}	2.31×10^{-03}	1.43×10^{-01}	7.85×10^{-14}	4.52×10^{-15}
Coding exons	0.00	0.00	0.00	1.86×10^{-27}	1.00	7.66×10^{-54}	1.05×10^{-113}	0.00	0.00
3’ UTR exons	1.59×10^{-218}	0.00	1.46×10^{-76}	2.31×10^{-03}	7.66×10^{-54}	1.00	1.37×10^{-02}	2.84×10^{-115}	9.49×10^{-118}
All exons	0.00	0.00	3.79×10^{-100}	1.43×10^{-01}	1.05×10^{-113}	1.37×10^{-02}	1.00	1.44×10^{-170}	2.58×10^{-171}
Introns	1.46×10^{-55}	1.78×10^{-129}	1.12×10^{-07}	7.85×10^{-14}	0.00	2.84×10^{-115}	1.44×10^{-170}	1.00	3.92×10^{-01}
Intragenic region	4.84×10^{-62}	1.66×10^{-132}	9.95×10^{-07}	4.52×10^{-15}	0.00	9.49×10^{-118}	2.58×10^{-171}	3.92×10^{-01}	1.00

Transition SNPs

	Intergenic region	CpG island	Promoter	5’ UTR exons	Coding exons	3’ UTR exons	All exons	Introns	Intragenic region
Intergenic region	1.00	1.31×10^{-07}	8.54×10^{-283}	4.59×10^{-206}	5.34×10^{-112}	6.29×10^{-215}	4.73×10^{-83}	3.07×10^{-32}	4.43×10^{-23}
CpG island	1.31×10^{-07}	1.00	1.54×10^{-61}	1.09×10^{-250}	3.54×10^{-43}	4.00×10^{-117}	5.19×10^{-13}	7.50×10^{-01}	2.87×10^{-02}
Promoter	8.54×10^{-283}	1.54×10^{-61}	1.00	4.77×10^{-97}	2.98×10^{-02}	7.44×10^{-17}	2.82×10^{-31}	2.92×10^{-156}	2.43×10^{-182}
5’ UTR exons	4.59×10^{-206}	1.09×10^{-250}	4.77×10^{-97}	1.00	5.37×10^{-121}	5.36×10^{-46}	4.51×10^{-156}	3.79×10^{-169}	9.25×10^{-191}
Coding exons	5.34×10^{-112}	3.54×10^{-43}	2.98×10^{-02}	5.37×10^{-121}	1.00	2.10×10^{-24}	1.93×10^{-14}	9.25×10^{-61}	3.71×10^{-77}
3’ UTR exons	6.29×10^{-215}	4.00×10^{-117}	7.44×10^{-17}	5.36×10^{-46}	2.10×10^{-24}	1.00	1.06×10^{-66}	1.92×10^{-142}	1.67×10^{-166}
All exons	4.73×10^{-83}	5.19×10^{-13}	2.82×10^{-31}	4.51×10^{-156}	1.93×10^{-14}	1.06×10^{-66}	1.00	1.07×10^{-30}	2.96×10^{-42}
Introns	3.07×10^{-32}	7.50×10^{-01}	2.92×10^{-156}	3.79×10^{-169}	9.25×10^{-61}	1.92×10^{-142}	1.07×10^{-30}	1.00	6.17×10^{-02}
Intragenic region	4.43×10^{-23}	2.87×10^{-02}	2.43×10^{-182}	9.25×10^{-191}	3.71×10^{-77}	1.67×10^{-166}	2.96×10^{-42}	6.17×10^{-02}	1.00

Transversion SNPs

	Intergenic region	CpG island	Promoter	5’ UTR exons	Coding exons	3’ UTR exons	All exons	Introns	Intragenic region
Intergenic region	1.00	1.23×10^{-139}	1.11×10^{-64}	1.85×10^{-206}	0.00	0.00	0.00	1.57×10^{-84}	6.33×10^{-106}
CpG island	1.23×10^{-139}	1.00	2.34×10^{-34}	4.37×10^{-226}	0.00	0.00	0.00	3.44×10^{-204}	9.99×10^{-214}
Promoter	1.11×10^{-64}	2.34×10^{-34}	1.00	2.00×10^{-281}	0.00	0.00	0.00	2.11×10^{-182}	7.41×10^{-201}
5’ UTR exons	1.85×10^{-206}	4.37×10^{-226}	2.00×10^{-281}	1.00	2.70×10^{-28}	2.72×10^{-26}	2.86×10^{-136}	4.27×10^{-175}	2.61×10^{-184}
Coding exons	0.00	0.00	0.00	2.70×10^{-28}	1.00	1.76×10^{-35}	3.15×10^{-221}	0.00	0.00
3’ UTR exons	0.00	0.00	0.00	2.72×10^{-26}	1.76×10^{-35}	1.00	4.46×10^{-28}	5.64×10^{-215}	2.92×10^{-216}
All exons	0.00	0.00	0.00	2.86×10^{-136}	3.15×10^{-221}	4.46×10^{-28}	1.00	2.68×10^{-274}	3.40×10^{-269}
Introns	1.57×10^{-84}	3.44×10^{-204}	2.11×10^{-182}	4.27×10^{-175}	0.00	5.64×10^{-215}	2.68×10^{-274}	1.00	2.89×10^{-02}
Intragenic region	6.33×10^{-106}	9.99×10^{-214}	7.41×10^{-201}	2.61×10^{-184}	0.00	2.92×10^{-216}	3.40×10^{-269}	2.89×10^{-02}	1.00

Indels

	Intergenic region	CpG island	Promoter	5’ UTR exons	Coding exons	3’ UTR exons	All exons	Introns	Intragenic region
Intergenic region	1.00	0.00	4.57×10^{-78}	0.00	0.00	0.00	0.00	1.08×10^{-29}	8.07×10^{-148}
CpG island	0.00	1.00	0.00	1.44×10^{-151}	9.51×10^{-247}	2.55×10^{-118}	2.41×10^{-145}	0.00	0.00
Promoter	4.57×10^{-78}	0.00	1.00	0.00	0.00	0.00	0.00	1.55×10^{-23}	7.96×10^{-01}
5’ UTR exons	0.00	1.44×10^{-151}	0.00	1.00	2.10×10^{-02}	0.00	0.00	0.00	0.00
Coding exons	0.00	9.51×10^{-247}	0.00	2.10×10^{-02}	1.00	0.00	0.00	0.00	0.00
3’ UTR exons	0.00	2.55×10^{-118}	0.00	0.00	0.00	1.00	9.06×10^{-08}	0.00	0.00
All exons	0.00	2.41×10^{-145}	0.00	0.00	0.00	9.06×10^{-08}	1.00	0.00	0.00
Introns	1.08×10^{-29}	0.00	1.55×10^{-23}	0.00	0.00	0.00	0.00	1.00	7.54×10^{-43}
Intragenic region	8.07×10^{-148}	0.00	7.96×10^{-01}	0.00	0.00	0.00	0.00	7.54×10^{-43}	1.00
