# Supplementary material for: SNP and indel frequencies at transcription start sites and at canonical and alternative translation initiation sites in the human genome
Source: PLoS One. 2019 Apr 12;14(4):e0214816. doi: 10.1371/journal.pone.0214816 (PMC6461226; doi:10.1371/journal.pone.0214816)
Supplement: S5 Table — Two-tailed Wilcoxon rank sum tests together with Bonferroni correction were used for the statistical comparison of the different SNP types within the nine genomic elements. Thereby, we assume a p-value to be significant if p < 1.4 × 10^−3^ which is equal to 0.05#tests where #tests=9×82. Note that due to numerical reasons, very small p–values (< 10−310) are represented as 0.0 in python programming language. (PDF) [file pone.0214816.s012.pdf]

S5 Table

**Intergenic region**				
	All variants	Transition SNPs	Transversion SNPs	Indels
All variants	1.00	0.00	0.00	0.00
Transition SNPs	0.00	1.00	0.00	0.00
Transversion SNPs	0.00	0.00	1.00	0.00
Indels	0.00	0.00	0.00	1.00
**CpG island**				
	All variants	Transition SNPs	Transversion SNPs	Indels
All variants	1.00	0.00	0.00	0.00
Transition SNPs	0.00	1.00	0.00	0.00
Transversion SNPs	0.00	0.00	1.00	0.00
Indels	0.00	0.00	0.00	1.00
**Promoter**				
	All variants	Transition SNPs	Transversion SNPs	Indels
All variants	1.00	0.00	0.00	0.00
Transition SNPs	0.00	1.00	0.00	0.00
Transversion SNPs	0.00	0.00	1.00	0.00
Indels	0.00	0.00	0.00	1.00
**5' UTR exons**				
	All variants	Transition SNPs	Transversion SNPs	Indels
All variants	1.00	1.77×10^{-195}	0.00	0.00
Transition SNPs	1.77×10^{-195}	1.00	3.58×10^{-127}	0.00
Transversion SNPs	0.00	3.58×10^{-127}	1.00	0.00
Indels	0.00	0.00	0.00	1.00
**Coding exons**				
	All variants	Transition SNPs	Transversion SNPs	Indels
All variants	1.00	0.00	0.00	0.00
Transition SNPs	0.00	1.00	0.00	0.00
Transversion SNPs	0.00	0.00	1.00	0.00
Indels	0.00	0.00	0.00	1.00
**3' UTR exons**				
	All variants	Transition SNPs	Transversion SNPs	Indels
All variants	1.00	0.00	0.00	0.00
Transition SNPs	0.00	1.00	0.00	0.00
Transversion SNPs	0.00	0.00	1.00	0.00
Indels	0.00	0.00	0.00	1.00
**All exons**				
	All variants	Transition SNPs	Transversion SNPs	Indels
All variants	1.00	0.00	0.00	0.00
Transition SNPs	0.00	1.00	0.00	0.00
Transversion SNPs	0.00	0.00	1.00	0.00
Indels	0.00	0.00	0.00	1.00
**Introns**				
	All variants	Transition SNPs	Transversion SNPs	Indels
All variants	1.00	0.00	0.00	0.00
Transition SNPs	0.00	1.00	0.00	0.00
Transversion SNPs	0.00	0.00	1.00	0.00
Indels	0.00	0.00	0.00	1.00
**Intragenic region**				
	All variants	Transition SNPs	Transversion SNPs	Indels
All variants	1.00	0.00	0.00	0.00
Transition SNPs	0.00	1.00	0.00	0.00
Transversion SNPs	0.00	0.00	1.00	0.00
Indels	0.00	0.00	0.00	1.00
