# Supplementary material for: SNP and indel frequencies at transcription start sites and at canonical and alternative translation initiation sites in the human genome
Source: PLoS One. 2019 Apr 12;14(4):e0214816. doi: 10.1371/journal.pone.0214816 (PMC6461226; doi:10.1371/journal.pone.0214816)
Supplement: S6 Table — Two-tailed Wilcoxon rank sum tests together with Bonferroni correction were used for the statistical comparison of the different SNP types within the nine genomic elements. Thereby, we assume a p-value to be significant if p < 1.4 × 10^−3^ which is equal to 0.05#tests where #tests=9×82. Note that due to numerical reasons, very small p–values (< 10−310) are represented as 0.0 in python programming language. (PDF) [file pone.0214816.s013.pdf]

S6 Table

All variants

	Intergenic region	CpG island	Promoter	5’ UTR exons	Coding exons	3’ UTR exons	All exons	Introns	Intragenic region
Intergenic region	1.00	2.88×10^{-191}	0.00	0.00	0.00	3.38×10^{-249}	0.00	2.48×10^{-79}	1.11×10^{-136}
CpG island	2.88×10^{-191}	1.00	1.00×10^{-08}	9.81×10^{-299}	3.33×10^{-220}	8.71×10^{-04}	8.36×10^{-18}	6.98×10^{-97}	2.45×10^{-76}
Promoter	0.00	1.00×10^{-08}	1.00	1.12×10^{-276}	0.00	3.95×10^{-08}	5.73×10^{-93}	3.13×10^{-142}	4.39×10^{-95}
5’ UTR exons	0.00	9.81×10^{-299}	1.12×10^{-276}	1.00	3.55×10^{-57}	3.32×10^{-170}	1.78×10^{-174}	0.00	0.00
Coding exons	0.00	3.33×10^{-220}	0.00	3.55×10^{-57}	1.00	2.06×10^{-199}	3.29×10^{-186}	0.00	0.00
3’ UTR exons	3.38×10^{-249}	8.71×10^{-04}	3.95×10^{-08}	3.32×10^{-170}	2.06×10^{-199}	1.00	1.59×10^{-21}	7.08×10^{-118}	2.68×10^{-88}
All exons	0.00	8.36×10^{-18}	5.73×10^{-93}	1.78×10^{-174}	3.29×10^{-186}	1.59×10^{-21}	1.00	0.00	0.00
Introns	2.48×10^{-79}	6.98×10^{-97}	3.13×10^{-142}	0.00	0.00	7.08×10^{-118}	0.00	1.00	1.41×10^{-09}
Intragenic region	1.11×10^{-136}	2.45×10^{-76}	4.39×10^{-95}	0.00	0.00	2.68×10^{-88}	0.00	1.41×10^{-09}	1.00

Transition SNPs

	Intergenic region	CpG island	Promoter	5’ UTR exons	Coding exons	3’ UTR exons	All exons	Introns	Intragenic region
Intergenic region	1.00	0.00	0.00	0.00	0.00	9.35×10^{-286}	0.00	9.37×10^{-61}	4.47×10^{-87}
CpG island	0.00	1.00	7.27×10^{-31}	0.00	6.44×10^{-01}	3.99×10^{-04}	2.17×10^{-50}	3.09×10^{-288}	2.21×10^{-279}
Promoter	0.00	7.27×10^{-31}	1.00	0.00	4.54×10^{-28}	2.30×10^{-01}	2.00×10^{-09}	0.00	0.00
5’ UTR exons	0.00	0.00	0.00	1.00	0.00	0.00	0.00	0.00	0.00
Coding exons	0.00	6.44×10^{-01}	4.54×10^{-28}	0.00	1.00	1.67×10^{-08}	9.47×10^{-54}	0.00	0.00
3’ UTR exons	9.35×10^{-286}	3.99×10^{-04}	2.30×10^{-01}	0.00	1.67×10^{-08}	1.00	1.26×10^{-09}	9.08×10^{-167}	1.27×10^{-153}
All exons	0.00	2.17×10^{-50}	2.00×10^{-09}	0.00	9.47×10^{-54}	1.26×10^{-09}	1.00	1.77×10^{-215}	8.20×10^{-190}
Introns	9.37×10^{-61}	3.09×10^{-288}	0.00	0.00	0.00	9.08×10^{-167}	1.77×10^{-215}	1.00	8.95×10^{-04}
Intragenic region	4.47×10^{-87}	2.21×10^{-279}	0.00	0.00	0.00	1.27×10^{-153}	8.20×10^{-190}	8.95×10^{-04}	1.00

Transversion SNPs

	Intergenic region	CpG island	Promoter	5’ UTR exons	Coding exons	3’ UTR exons	All exons	Introns	Intragenic region
Intergenic region	1.00	1.26×10^{-12}	1.60×10^{-01}	0.00	0.00	0.00	0.00	5.57×10^{-106}	3.37×10^{-182}
CpG island	1.26×10^{-12}	1.00	5.00×10^{-20}	0.00	0.00	2.74×10^{-120}	3.83×10^{-89}	9.65×10^{-01}	3.84×10^{-02}
Promoter	1.60×10^{-01}	5.00×10^{-20}	1.00	0.00	0.00	0.00	0.00	1.98×10^{-37}	3.23×10^{-70}
5’ UTR exons	0.00	0.00	0.00	1.00	5.64×10^{-193}	2.78×10^{-201}	0.00	0.00	0.00
Coding exons	0.00	0.00	0.00	5.64×10^{-193}	1.00	1.26×10^{-73}	4.99×10^{-274}	0.00	0.00
3’ UTR exons	0.00	2.74×10^{-120}	0.00	2.78×10^{-201}	1.26×10^{-73}	1.00	1.72×10^{-13}	1.56×10^{-274}	1.71×10^{-243}
All exons	0.00	3.83×10^{-89}	0.00	0.00	4.99×10^{-274}	1.72×10^{-13}	1.00	0.00	0.00
Introns	5.57×10^{-106}	9.65×10^{-01}	1.98×10^{-37}	0.00	0.00	1.56×10^{-274}	0.00	1.00	2.47×10^{-11}
Intragenic region	3.37×10^{-182}	3.84×10^{-02}	3.23×10^{-70}	0.00	0.00	1.71×10^{-243}	0.00	2.47×10^{-11}	1.00

Indels

	Intergenic region	CpG island	Promoter	5’ UTR exons	Coding exons	3’ UTR exons	All exons	Introns	Intragenic region
Intergenic region	1.00	0.00	1.97×10^{-234}	0.00	0.00	0.00	0.00	6.26×10^{-02}	7.08×10^{-43}
CpG island	0.00	1.00	0.00	8.68×10^{-34}	1.25×10^{-10}	0.00	0.00	0.00	0.00
Promoter	1.97×10^{-234}	0.00	1.00	0.00	0.00	6.64×10^{-95}	3.65×10^{-251}	1.00×10^{-180}	1.99×10^{-104}
5’ UTR exons	0.00	8.68×10^{-34}	0.00	1.00	1.38×10^{-09}	0.00	0.00	0.00	0.00
Coding exons	0.00	1.25×10^{-10}	0.00	1.38×10^{-09}	1.00	0.00	0.00	0.00	0.00
3’ UTR exons	0.00	0.00	6.64×10^{-95}	0.00	0.00	1.00	5.77×10^{-05}	0.00	0.00
All exons	0.00	0.00	3.65×10^{-251}	0.00	0.00	5.77×10^{-05}	1.00	0.00	0.00
Introns	6.26×10^{-02}	0.00	1.00×10^{-180}	0.00	0.00	0.00	0.00	1.00	1.10×10^{-28}
Intragenic region	7.08×10^{-43}	0.00	1.99×10^{-104}	0.00	0.00	0.00	0.00	1.10×10^{-28}	1.00
