# Supplementary material for: SNP and indel frequencies at transcription start sites and at canonical and alternative translation initiation sites in the human genome
Source: PLoS One. 2019 Apr 12;14(4):e0214816. doi: 10.1371/journal.pone.0214816 (PMC6461226; doi:10.1371/journal.pone.0214816)
Supplement: S7 Table — Shown are the Top100 genes with the largest SNP densities from the European cohort of the 1000G set by sorting with respect to coding exons. (PDF) [file pone.0214816.s014.pdf]

S7 Table

	Gene name	Element length [kb]	Nbr. of all SNPs	SNP density
1	HLADQB1	0.81	71	87.65
2	HLADQA1	0.768	67	87.24
3	HLADRB5	0.801	68	84.89
4	CRIPAK	1.341	92	68.61
5	IGLL5	0.645	35	54.26
6	PRR21	1.17	55	47.01
7	PRAMEF2	1.425	61	42.81
8	KRTAP48	0.558	22	39.43
9	PLIN4	4.074	159	39.03
10	MUC4	16.239	594	36.58
11	CCDC144NL	0.666	24	36.04
12	DEFB108B	0.222	8	36.04
13	IGLL1	0.642	23	35.83
14	OR2T8	0.939	33	35.14
15	ABO	1.064	37	34.77
16	HRCT1	0.348	12	34.48
17	HIST1H1C	0.642	22	34.27
18	KRTAP47	0.468	16	34.19
19	KRTAP411	0.588	20	34.01
20	SLC35G4	1.017	34	33.43
21	KIR3DL1	1.335	44	32.96
22	GGTLC2	0.759	25	32.94
23	OR5H6	0.978	31	31.7
24	KIR2DL1	1.047	33	31.52
25	OR51F1	0.939	29	30.88
26	IGIP	0.162	5	30.86
27	FAM90A1	1.395	43	30.82
28	CELA3B	0.813	25	30.75
29	C9orf66	0.888	27	30.41
30	FAM86C1	0.396	12	30.3
31	KRTAP32	0.297	9	30.3
32	OR8U1	0.93	27	29.03
33	KRTAP96	0.483	14	28.99
34	HIST1H2BF	0.381	11	28.87
35	HIST1H1E	0.66	19	28.79
36	HIST1H2BA	0.384	11	28.65
37	HIST1H1D	0.666	19	28.53
38	PDIA2	1.578	45	28.52
39	GPRIN2	1.377	39	28.32
40	PI3	0.354	10	28.25
41	PSG9	1.281	36	28.1
42	DEFA5	0.285	8	28.07
43	OR5H14	0.933	26	27.87
44	SCGB1C1	0.288	8	27.78
45	OR2T4	1.047	29	27.7
46	AHNAK2	17.388	480	27.61
47	PRAMEF10	1.425	39	27.37
48	LILRA1	1.47	40	27.21
49	OR13C5	0.957	26	27.17
50	HGC63	0.516	14	27.13

	Gene name	Element length [kb]	Nbr. of all SNPs	SNP density
51	LCE2B	0.333	9	27.03
52	CPZ	1.926	52	27.0
53	CYP2A7	1.485	40	26.94
54	CD52	0.186	5	26.88
55	KRTAP49	0.633	17	26.86
56	HS3ST6	0.936	25	26.71
57	MTRNR2L5	0.075	2	26.67
58	MTRNR2L9	0.075	2	26.67
59	TRIM48	0.675	18	26.67
60	HIGD1B	0.3	8	26.67
61	LILRB1	1.953	52	26.63
62	TPSB2	0.828	22	26.57
63	MRPL53	0.339	9	26.55
64	INMT	0.792	21	26.52
65	MT4	0.189	5	26.46
66	OR2T27	0.954	25	26.21
67	LILRA2	1.452	38	26.17
68	OR6K6	1.032	27	26.16
69	S100A7A	0.306	8	26.14
70	CYP2D6	1.341	35	26.1
71	LOC100132146	0.27	7	25.93
72	TMEM244	0.387	10	25.84
73	LILRA3	1.32	34	25.76
74	USP17L7	1.593	41	25.74
75	ACTG1	1.128	29	25.71
76	HIST1H4B	0.312	8	25.64
77	PRM1	0.156	4	25.64
78	OR10G9	0.936	24	25.64
79	HIST1H4J	0.312	8	25.64
80	C20orf166	0.354	9	25.42
81	OBP2A	0.513	13	25.34
82	PTX4	1.422	36	25.32
83	FLG	12.186	308	25.27
84	LCE1E	0.357	9	25.21
85	MC1R	0.954	24	25.16
86	PSG6	1.275	32	25.1
87	LCE3D	0.279	7	25.09
88	LRP5L	0.759	19	25.03
89	HIST1H1B	0.681	17	24.96
90	C14orf180	0.483	12	24.84
91	MS4A13	0.282	7	24.82
92	CCL22	0.282	7	24.82
93	OR2T6	0.927	23	24.81
94	AMZ1	1.497	37	24.72
95	LOC100134391	0.891	22	24.69
96	ALG1L2	0.648	16	24.69
97	DAPL1	0.324	8	24.69
98	PSG1	1.26	31	24.6
99	KRTAP14	0.366	9	24.59
100	OR10G4	0.936	23	24.57
