# Supplementary material for: SNP and indel frequencies at transcription start sites and at canonical and alternative translation initiation sites in the human genome
Source: PLoS One. 2019 Apr 12;14(4):e0214816. doi: 10.1371/journal.pone.0214816 (PMC6461226; doi:10.1371/journal.pone.0214816)
Supplement: S8 Table — Shown are the Top100 genes with the largest SNP densities from the European cohort of the 1000G set by sorting with respect to promoter regions. (PDF) [file pone.0214816.s015.pdf]

S8 Table

	Gene name	Element length [kb]	Nbr. of all SNPs	SNP density
1	HLADQA1	3.0	300	100.0
2	HLADQB1	3.0	299	99.67
3	IGLL5	3.0	189	63.0
4	PSG4	3.0	143	47.67
5	MTRNR2L1	3.0	132	44.0
6	PSG7	3.0	121	40.33
7	BCL6	3.0	116	38.67
8	PSG11	3.0	112	37.33
9	HLADRB5	3.0	103	34.33
10	PSG5	3.0	101	33.67
11	PSG6	3.0	97	32.33
12	PSG8	3.0	93	31.0
13	WDR1	3.0	91	30.33
14	TMEM14C	3.0	89	29.67
15	OR11H4	3.0	89	29.67
16	FDFT1	3.0	88	29.33
17	CD300A	3.0	86	28.67
18	WDR74	3.0	86	28.67
19	USP17L7	3.0	85	28.33
20	CCDC144NL	3.0	82	27.33
21	TUBA3C	3.0	79	26.33
22	PRR21	3.0	79	26.33
23	USP17L2	3.0	78	26.0
24	CGB	3.0	76	25.33
25	ORC5	3.0	76	25.33
26	KRTAP48	3.0	76	25.33
27	PRLH	3.0	75	25.0
28	HIST1H2BA	3.0	75	25.0
29	OR2T27	3.0	74	24.67
30	DEF8	3.0	73	24.33
31	ZNF16	3.0	73	24.33
32	JAKMIP3	3.0	72	24.0
33	KIR2DS4	3.0	71	23.67
34	ZFAND2A	3.0	71	23.67
35	D2HGDH	3.0	70	23.33
36	OR13C5	3.0	69	23.0
37	HIST1H2AA	3.0	68	22.67
38	PSG9	3.0	68	22.67
39	PLEKHN1	3.0	68	22.67
40	POLDIP3	3.0	67	22.33
41	FRMD1	3.0	67	22.33
42	OR51F1	3.0	67	22.33
43	PGPEP1L	3.0	67	22.33
44	KRTAP98	3.0	67	22.33
45	TCL1A	3.0	66	22.0
46	FAM160B2	3.0	66	22.0
47	PSG1	3.0	66	22.0
48	TRMT10B	3.0	66	22.0
49	C12orf57	3.0	66	22.0
50	MCPH1	3.0	66	22.0

	Gene name	Element length [kb]	Nbr. of all SNPs	SNP density
51	SERPINA9	3.0	66	22.0
52	TUSC5	3.0	66	22.0
53	CGB7	3.0	66	22.0
54	C19orf10	3.0	66	22.0
55	FCGR3B	3.0	65	21.67
56	TTI2	3.0	65	21.67
57	PRTN3	3.0	65	21.67
58	DEFB1	3.0	65	21.67
59	SLC35G4	3.0	65	21.67
60	LILRB2	3.0	65	21.67
61	RFPL3	3.0	65	21.67
62	TMEM88B	3.0	65	21.67
63	GCC2	3.0	64	21.33
64	SSR1	3.0	64	21.33
65	TLDC1	3.0	64	21.33
66	PSG2	3.0	64	21.33
67	KRT5	3.0	64	21.33
68	IFITM2	3.0	63	21.0
69	CES1	3.0	63	21.0
70	ALG12	3.0	63	21.0
71	AQP12B	3.0	63	21.0
72	ZNF761	3.0	63	21.0
73	HIST1H2BF	3.0	63	21.0
74	IRGM	3.0	63	21.0
75	ZNF57	3.0	63	21.0
76	OR5H6	3.0	63	21.0
77	MSLN	3.0	63	21.0
78	CYP4F12	3.0	62	20.67
79	HIST1H4H	3.0	62	20.67
80	GZMM	3.0	62	20.67
81	PSG3	3.0	62	20.67
82	C1QTNF8	3.0	62	20.67
83	MS4A12	3.0	61	20.33
84	ZNF812	3.0	61	20.33
85	AMTN	3.0	61	20.33
86	ZNF773	3.0	61	20.33
87	CRELD2	3.0	61	20.33
88	SLC35G5	3.0	61	20.33
89	PLCG2	3.0	61	20.33
90	HIST1H2AC	3.0	61	20.33
91	TMEM105	3.0	61	20.33
92	TREML4	3.0	61	20.33
93	ZNF443	3.0	61	20.33
94	SLC22A16	3.0	61	20.33
95	XCL2	3.0	61	20.33
96	TTLL10	3.0	61	20.33
97	DHRS4	3.0	60	20.0
98	ZNF320	3.0	60	20.0
99	CD7	3.0	60	20.0
100	RPUSD1	3.0	60	20.0
