# Supplementary material for: SNP and indel frequencies at transcription start sites and at canonical and alternative translation initiation sites in the human genome
Source: PLoS One. 2019 Apr 12;14(4):e0214816. doi: 10.1371/journal.pone.0214816 (PMC6461226; doi:10.1371/journal.pone.0214816)
Supplement: S9 Table — Results of DAVID functional annotation [48] for all genes that contain such dinucleotides at TSS position −1. Duplicated terms from different databases were deleted and the one with smallest p–value was retained. Shown are terms with corrected p–value of p < 0.05 (Benjamini correction). If no significant GO term enrichment was found for a dinucleotide gene subset, only the first two terms are displayed for convenience. The number of genes (RefSeq identifiers accepted by DAVID tool) of every subgroup is given in brackets. (PDF) [file pone.0214816.s016.pdf]

S9 Table

		Term	# Genes	% Genes	Adjusted p–value
**ApA (530 genes)**	1.	Detection of chemical stimulus involved in sensory perception of smell	97	18.3	1.4×10^{-61}
	2.	Olfactory receptor	97	18.3	1.1×10^{-61}
	3.	Olfactory receptor activity	97	18.3	3.5×10^{-61}
	4.	Olfaction	97	18.3	7.1×10^{-61}
	5.	Olfactory transduction	97	18.3	3.4×10^{-53}
	6.	Sensory transduction	103	19.4	5.8×10^{-52}
	7.	G-protein coupled receptor activity	108	20.4	1.6×10^{-49}
	8.	GPCR, rhodopsin–like, 7TM	105	19.8	1.5×10^{-46}
	9.	G–protein coupled receptor	112	21.1	1.5×10^{-46}
	10.	G–protein coupled receptor, rhodopsin–like	103	19.4	6.2×10^{-46}
	11.	Transducer	113	21.3	2.4×10^{-44}
	12.	G-protein coupled receptor signaling pathway	112	21.1	7.4×10^{-42}
	13.	Receptor	132	24.9	4.6×10^{-29}
	14.	Topological domain: extracellular	161	30.4	9.4×10^{-20}
	15.	Disulfide bond	162	30.6	2.8×10^{-18}
	16.	Topological domain: cytoplasmic	179	33.8	1.3×10^{-17}
	17.	Glycosylation site: N–linked	202	38.1	3.9×10^{-16}
	18.	Odorant binding	26	4.9	3.3×10^{-16}
	19.	Disulfide bond	173	32.6	2.8×10^{-16}
	20.	Glycoprotein	209	39.4	1.1×10^{-14}
	21.	Cell membrane	156	29.4	3.5×10^{-13}
	22.	Detection of chemical stimulus involved in sensory perception	22	4.2	1.2×10^{-11}
	23.	Plasma membrane	184	34.7	2.7×10^{-11}
	24.	Sensory perception of smell	24	4.5	1.4×10^{-9}
	25.	Transmembrane region	202	38.1	1.0×10^{-8}
	26.	Transmembrane helix	216	40.8	1.8×10^{-8}
	27.	Integral component of membrane	199	37.5	9.1×10^{-8}
	28.	Transmembrane	216	40.8	2.2×10^{-8}
	29.	Transmembrane signaling receptor activity	22	4.2	6.1×10^{-5}
	30.	Membrane	255	48.1	4.3×10^{-5}
**ApC (392 genes)**	1.	Extracellular space	45	11.5	3.6×10^{-1}
	2.	Biosynthesis of antibiotics	12	3.1	2.8×10^{-1}
**ApG (1,011 genes)**	1.	Domain: Leucine–zipper	17	1.7	5.7×10^{-1}
	2.	DNA-binding region: basic motif	20	2.0	9.5×10^{-1}
**ApT (223 genes)**	1.	Commissural neuron axon guidance	3	1.3	9.8×10^{-1}
	2.	Disulfide bond	55	24.7	7.6×10^{-1}
