# Supplementary material for: SNP and indel frequencies at transcription start sites and at canonical and alternative translation initiation sites in the human genome
Source: PLoS One. 2019 Apr 12;14(4):e0214816. doi: 10.1371/journal.pone.0214816 (PMC6461226; doi:10.1371/journal.pone.0214816)
Supplement: S10 Table — Results of DAVID functional annotation [48] for all genes that contain such dinucleotides at TSS position −1. Duplicated terms from different databases were deleted and the one with smallest p–value was retained. Shown are terms with corrected p–value of p < 0.05 (Benjamini correction). If no significant GO term enrichment was found for a dinucleotide gene subset, only the first two terms are displayed for convenience. The number of genes (RefSeq identifiers accepted by DAVID tool) of every subgroup is given in brackets. (PDF) [file pone.0214816.s017.pdf]

S10 Table

		Term	# Genes	% Genes	Adjusted p–value
**CpA (3,412 genes)**	1.	Topological domain: extracellular	587	17.2	1.9×10^{-5}
	2.	G–protein coupled receptor signaling pathway	213	6.2	1.8×10^{-4}
	3.	Olfactory receptor activity	112	3.3	7.5×10^{-5}
	4.	GPCR, rhodopsin–like, 7TM	174	5.1	1.5×10^{-4}
	5.	G–protein coupled receptor, rhodopsin-like	170	5.0	1.0×10^{-4}
	6.	Olfactory receptor	112	3.3	7.4×10^{-5}
	7.	Detection of chemical stimulus involved in sensory perception of smell	111	3.3	2.5×10^{-4}
	8.	Olfaction	114	3.3	5.4×10^{-5}
	9.	Topological domain: cytoplasmic	701	20.5	3.7×10^{-4}
	10.	Transmembrane region	981	28.8	2.5×10^{-4}
	11.	Transducer	207	6.1	5.3×10^{-5}
	12.	G–protein coupled receptor	194	5.7	5.4×10^{-5}
	13.	Glycosylation site: N–linked (GlcNAc...)	844	24.7	8.1×10^{-4}
	14.	Olfactory transduction	112	3.3	2.5×10^{-4}
	15.	Integral component of membrane	989	29.0	1.0×10^{-3}
	16.	Glycoprotein	906	26.6	1.9×10^{-4}
	17.	Sensory transduction	146	4.3	1.9×10^{-4}
	18.	G-protein coupled receptor activity	163	4.8	1.7×10^{-3}
	19.	Transmembrane	1076	31.5	2.5×10^{-4}
	20.	Transmembrane helix	1072	31.4	2.4×10^{-4}
	21.	Cell membrane	635	18.6	4.1×10^{-4}
	22.	Receptor	350	10.3	6.8×10^{-4}
	23.	Nucleosome core	31	0.9	1.6×10^{-3}
	24.	Sensory perception of smell	44	1.3	8.6×10^{-2}
	25.	Histone–fold	35	1.0	4.0×10^{-2}
	26.	Keratin–associated matrix	11	0.3	3.6×10^{-2}
	27.	Disulfide bond	670	19.6	3.6×10^{-3}
	28.	TAF	10	0.3	4.1×10^{-2}
	29.	H4	10	0.3	4.1×10^{-2}
	30.	TATA box binding protein associated factor (TAF)	10	0.3	3.9×10^{-2}
	31.	Histone H4	10	0.3	3.9×10^{-2}
**CpC (1,289 genes)**	1.	Mitochondrion	119	9.2	3.6×10^{-1}
	2.	Neuron projection	30	2.3	5.0×10^{-1}
**CpG (2,136 genes)**	see Table 2 in the main manuscript				
**CpT (625 genes)**	1.	Signal	153	24.5	7.1×10^{-1}
	2.	Secreted	84	13.4	4.8×10^{-1}
