# Supplementary material for: SNP and indel frequencies at transcription start sites and at canonical and alternative translation initiation sites in the human genome
Source: PLoS One. 2019 Apr 12;14(4):e0214816. doi: 10.1371/journal.pone.0214816 (PMC6461226; doi:10.1371/journal.pone.0214816)
Supplement: S11 Table — Results of DAVID functional annotation [48] for all genes that contain such dinucleotides at TSS position −1. Duplicated terms from different databases were deleted and the one with smallest p–value was retained. Shown are terms with corrected p–value of p < 0.05 (Benjamini correction). If no significant GO term enrichment was found for a dinucleotide gene subset, only the first two terms are displayed for convenience. The number of genes (RefSeq identifiers accepted by DAVID tool) of every subgroup is given in brackets. (PDF) [file pone.0214816.s018.pdf]

S11 Table

		Term	# Genes	% Genes	Adjusted p–value
**GpA (1,005 genes)**	1.	G–protein coupled receptor activity	61	6.1	9.0×10^{-3}
	2.	G–protein coupled receptor, rhodopsin–like	60	6.0	4.7×10^{-2}
	3.	G-protein coupled receptor	68	6.8	2.0×10^{-2}
	4.	Odorant binding	16	1.6	2.4×10^{-2}
	5.	GPCR, rhodopsin–like, 7TM	60	6.0	4.8×10^{-2}
**GpC (871 genes)**	1.	Calcium transport	16	1.8	4.0×10^{-2}
**GpG (1,413 genes)**	1.	Splice variant	637	45.1	3.2×10^{-2}
**GpT (282 genes)**	1.	IL12 and Stat4 Dependent Signaling Pathway in Th1 Development	4	1.4	1.6×10^{-1}
	2.	snRNA processing	3	1.1	1.0
