# Supplementary material for: SNP and indel frequencies at transcription start sites and at canonical and alternative translation initiation sites in the human genome
Source: PLoS One. 2019 Apr 12;14(4):e0214816. doi: 10.1371/journal.pone.0214816 (PMC6461226; doi:10.1371/journal.pone.0214816)
Supplement: S12 Table — Results of DAVID functional annotation [48] for all genes that contain such dinucleotides at TSS position −1. Duplicated terms from different databases were deleted and the one with smallest p–value was retained. Shown are terms with corrected p–value of p < 0.05 (Benjamini correction). If no significant GO term enrichment was found for a dinucleotide gene subset, only the first two terms are displayed for convenience. The number of genes (RefSeq identifiers accepted by DAVID tool) of every subgroup is given in brackets. (PDF) [file pone.0214816.s019.pdf]

S12 Table

		Term	# Genes	% Genes	Adjusted p–value
**TpA (951 genes)**	1.	Olfaction	49	5.2	6.1×10^{-6}
	2.	Olfactory receptor activity	48	5.0	1.5×10^{-5}
	3.	Detection of chemical stimulus involved in sensory perception of smell	48	5.0	5.0×10^{-5}
	4.	Olfactory receptor	48	5.0	4.1×10^{-5}
	5.	Olfactory transduction	50	5.3	1.3×10^{-5}
	6.	Sensory transduction	58	6.1	8.5×10^{-5}
	7.	G–protein coupled receptor activity	59	6.2	7.2×10^{-3}
**TpC (698 genes)**	1.	Transcription factor activity, sequence–specific DNA binding	59	8.5	1.0×10^{-1}
	2.	DNA–binding	100	14.3	7.1×10^{-1}
**TpG (1,519 genes)**	1.	Xenobiotic metabolic process	16	1.1	9.7×10^{-1}
	2.	Protease inhibitor	20	1.3	8.2×10^{-1}
**TpT (238 genes)**	1.	Kinetochore binding	3	1.3	3.4×10^{-1}
	2.	Negative regulation of transcription, DNA–templated	16	6.7	7.6×10^{-1}
